# Potential reduction of Hartweg´s Pine (*Pinus hartwegii Lindl*.) geographic distribution

**DOI:** 10.1371/journal.pone.0229178

**Published:** 2020-02-18

**Authors:** Farid Uriel Alfaro-Ramírez, Jorge Enrique Ramírez-Albores, J. Jesús Vargas-Hernández, Sergio Franco-Maass, Marlín Pérez-Suárez

**Affiliations:** 1 Instituto de Ciencias Agropecuarias y Rurales (ICAR), Universidad Autónoma del Estado de México, El Cerrillo Piedras Blancas, Toluca de Lerdo, Estado de México, México; 2 Museo de Zoología “Alfonso L. Herrera”, Departamento de Biología Evolutiva, Facultad de Ciencias, Universidad Nacional Autónoma de México, Ciudad de México, México; 3 Colegio de Postgraduados, Campus Montecillo, Carretera México-Texcoco, Montecillo, Estado de México, México; Universidade Federal de Goias, BRAZIL

## Abstract

Geographical distribution of forest species is closely regulated by environmental conditions, particularly temperature and precipitation. Climate change predicted by general circulation models is expected to modify the distribution of many species’ distribution, especially those adapted to extreme environmental conditions, leading to large-scale migrations or local extinctions. The aim of this research was to determine the potential impact of climatic change on *Pinus hartwegii* geographic distribution and the niche breadth of its populations. Ecological niche models were used by generated with four different algorithms based on 19 bioclimatic variables in addition to altitude. Climatic niche breadth was delimited by the dispersion of species occurrence records within the intervals of the bioclimatic variables. We modelled future distribution based on three general circulation models, MIROC-ESM-CHEM, CCSM4 and HadGEM2-ES, using two representative concentration pathways (RCP) 2.6 and 8.5, for two-time horizons 2050 and 2070. Niche breadth analysis showed narrow ranges of suitability, indicating a strong relationship between the presence of *P*. *hartwegii* with the temperature of the warmest quarter and precipitation of the coldest quarter. In addition, the suitability area of *P*. *hartwegii* is predicted to be reduced up to 70% by 2070; the populations of the extreme northern and southern latitudes will be reduced in greater proportion than those of central Mexico. This suggest that environmental suitability area of *P*. *hartwegii* are reduced by the effect of the increase in environmental temperature. Therefore, it is necessary to monitor extreme populations of this species in the long term in order to establish efficient conservation strategies and well adaptive management facing climate change.

## Introduction

Climate is the main determining factor of plant species distribution on a global scale [1]. At local level, however, the plant species presence depends not only on climate but also on other factors such as altitude, availability of microsites and biological interactions [2, 3]. There is therefore some environmental specificity in the presence of a stable population of a particular species [4]. Geographical distribution of species is not random [5], and their dispersal and adaptability depend on the intrinsic limitations of the species. In addition, these which could be accentuated by ecological and physiological aspects specific of these species, such as biological associations, growth rate, or the pollen and seeds dispersion [6, 7], which aspects that can be drastically modified by changes in climatic conditions [1, 8, 9]. Moreover, to the complex interaction of limiting environmental factors as temperature or precipitation [10]. Therefore, each species occupies a specific area that represents a unique niche in the environment [4], and that persists along its evolutionary lineage [3]. However, when a modification of environmental condition resulted from some natural or anthropic perturbation the species could have conservative, breadth or shift its niche as an evolutive response. Under context of niche conservatism, the potential altitudinal migration of many forest species in the limit of its altitudinal distribution could result from the need of the species to conservative its niche. This last refers to the retention of certain characteristics of the ancestral fundamental niche over time and space [10]. However, the species also could breadth its niche adapting to the new conditions or even change it. To know the capacity of one species to conserve its niche is particularly important to predict future variations in the geographical distribution of a species as effect of climatic change [11], as the projections are based on the central hypothesis of niche conservatism [4, 12]. Accordingly, it has been suggested that if the climatic tolerance of a species is not extensive enough to face new environmental conditions, then those species with strong niche conservatism must migrate or become extinct [10, 13]. Climate change could significantly accelerate the migration or potential reduction of forest populations [8, 14, 15], due to the alteration of regulatory variables such as temperature, precipitation or wind intensity [8, 9]. Another important aspect is the genetic variability reduction of some populations, which limits their plasticity to adapt to new conditions [16, 17, 18] and then limit the survival of these species or their establishment at higher altitudes or northern latitudes [11, 14].

Species adapted to temperate and cold climates are more susceptible to climate change effects [3, 9]. Species distributed at high altitudes [7, 16, 19] are particularly are subject to harsh conditions such as shallow soils, low CO_2_ partial pressure or high radiation indices [2, 8]. In this sense, there are reports that associate the increase in temperature with the reduction of the area occupied by forest species such as the Mexican White Pine (*Pinus ayacahuite*) [15] and the False Weymouth Pine (*Pinus pseudostrobus*) [14]; others predict massive migrations of Egg-Cone Pine (*Pinus oocarpa*) populations [20], or the growth rate reduction of Hartweg's Pine (*Pinus hartwegii*) [21]. Some projections even point to the local extinction of these and other associated species [22], which could be accentuated by the increase in pest incidence associated with temperature increase [8]. Under this vision, several models have been generated to evaluate the potential impact of climate change on forest species [23], producing a wide variety of projections that seek to represent the possible responses of these species [24]. In general, the most realistic models are those that include vegetation dynamics and atmospheric chemistry [23], due to these factors improve the model’s predictive quality, even under different future scenarios. For Mexico, an increase of between 2 and 4°C is predicted for the 2020–2080 period [25]. Under this scenario, the exclusive habitat of species adapted to high mountain conditions could be reduced or even disappear [14, 15], due the fact that the ideal conditions for these species could be located at higher altitudes or northern latitudes in the next 50 years [26].

Hartweg´s Pine (*Pinus hartwegii* Lindl.) is considered one of the most vulnerable species to climate change [14, 15]. It has a discontinuous geographic distribution, from northeastern Mexico to northern El Salvador [27]. *Pinus hartwegii* presence is restricted to the highest Mexican peaks, mainly in the Transmexican Volcanic Belt, such as Nevado de Colima, Nevado de Toluca, Popocatepetl-Iztaccihuatl, Pico de Orizaba and Cofre de Perote [28, 29, 30]. Previous studies [14, 15, 31], agree that the occupied area by this species in central Mexico will be reduced between 10% and 70% in response to climate change or even reaching the extinction of some of its population [1, 9]. The foregoing is of great local and regional importance because this species is tolerant to the low temperatures that dominate the high altitudes, being able to form forest that reach the limit of the tree line up to 4,200 m a.s.l. [29]. Therefore, this forest ecosystems are essential to regional climate regulation and others ecosystem services such as wind regulation, water harvesting, carbon sequestration and wildlife refuges throughout its geographical distribution [6, 25, 26, 32]. Despite the great importance of this species, the optimal intervals of climatic variables in which the species is found (i.e., the climatic niche associated with *P*. *hartwegii*) have not been studied. For this reason, knowledge of the potential distribution and niche breadth of this species could contribute understand the long-term effects of temperature increase and changes in precipitation regimes on their populations. Therefore, the objective of this research was to determine the potential impact of climate change on the distribution of *P*. *hartwegii* populations and their niche breadth within the territory occupied by this species under different climate change scenarios. This information will allow long-term monitoring of the distribution of *P*. *hartwegii* populations, and the identification of risk areas and opportunities for the conservation of this species in the face of the effects of climate change.

## Materials and methods

### Occurrence records

**Fig 1:**
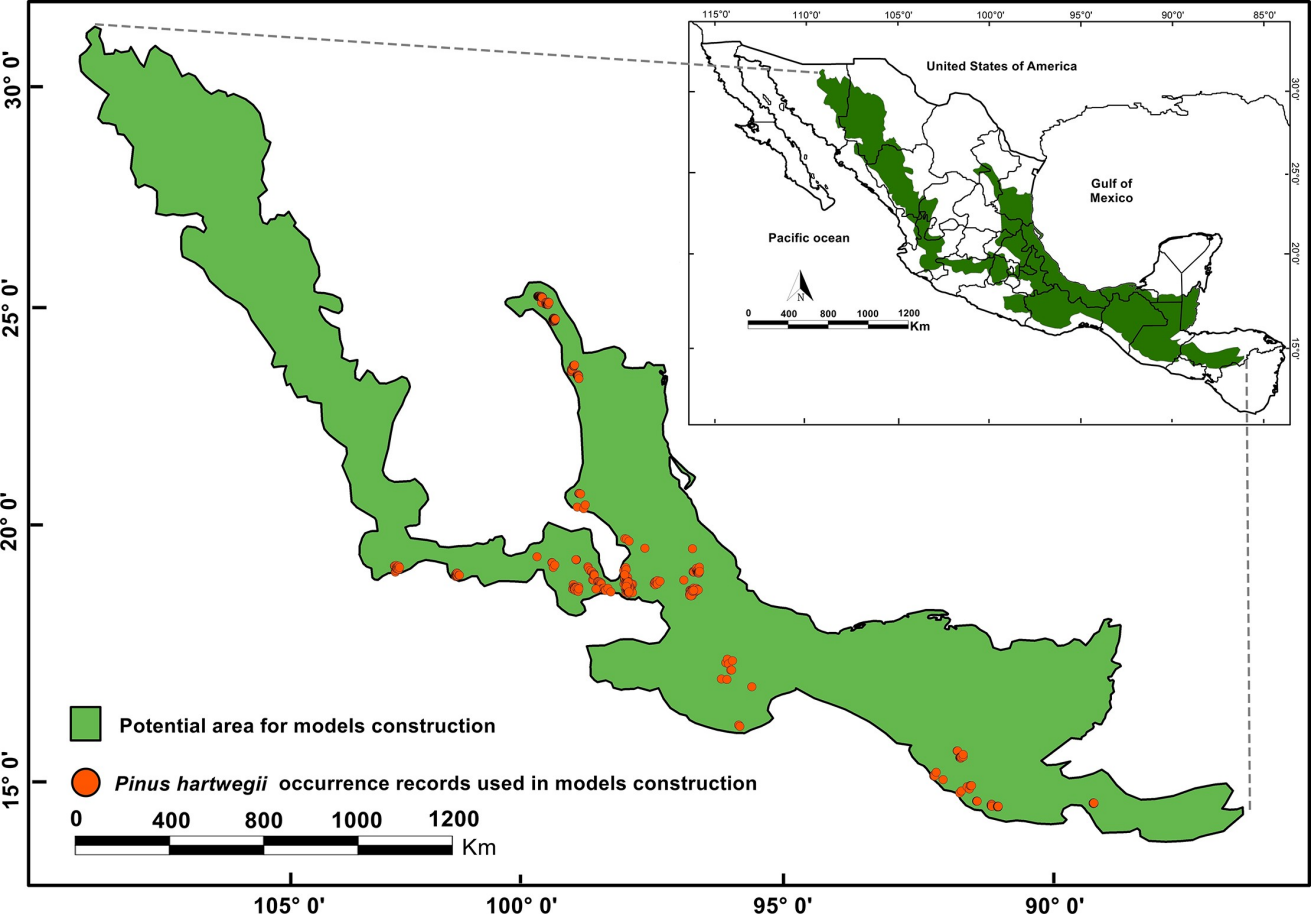
*Pinus hartwegii* current geographic distribution (shaded areas) and occurrence point used in the construction of its climate niche model (dots show).

*Pinus hartwegii* occurrence data throughout its geographical distribution (from Mexico to Central America) were collected through GBIF (download 23 February 2017 and available at https://www.gbif.org/), as well as from specimens of Mexican herbaria as Instituto de Biología-UNAM (MEXU), Escuela Nacional de Ciencias Biológicas-IPN (ENCB), Instituto de Ecología A.C. (XAL), Universidad Veracruzana Campus Xalapa (XALU), Universidad Veracruzana Campus Córdoba (CORU), and Colegio de Postgraduados Campus Montecillo (CHAPA). In addition, online databases of international herbaria such as the New York Botanical Garden (NYBG; http://sweetgum.nybg.org/science/vh/), Missouri Botanical Garden (MBG; http://www.tropicos.org/Home.aspx), Academy of Natural Sciences of Philadelphia (ANSP; http://ph.ansp.org/), and the Field Museum of Natural History (FMNH; http://fm1.fieldmuseum.org/vrrc/index.php?) were consulted. The database was complemented with records obtained in the field between September 2014 and August 2017 from Pico de Orizaba, La Malinche, Iztaccíhuatl-Popocatepetl, and Cerro El Potosi, which were obtained with a Garmin GPS (Model V, USA). *Pinus hartwegii* occurrence data were recorded by systematic stratified sampling to reduce the number of occurrence points per pixel. In addition, the widest possible range of geographic conditions under which the species is distributed was used, improving the homogeneity of the sample and reducing the prevalence of the species itself in a given set of environmental characteristics, in order to reduce the final bias in the suitability model [4]. This sampling was performed on the database obtained that represents the known distribution of the species and was used as background area, which corresponds to the distribution range 14° to 25° N and -97° to -103° W (Fig 1). A total of 1,788 occurrence records were obtained for *P*. *hartwegii*. This database was refined by (i) removing records prior to 1980 by the advanced of agriculture, (ii) duplicate occurrence records, (iii) occurrence records with coordinates outside of the natural distribution of the species and, (iv) occurrence records that did not have specific coordinates using package CoordinateCleaner in R-Studio [33]. Once the database was cleaned up, a total of 477 unique occurrence records were obtained, that is, the records represent a unique location in a 30" of arc grid (approximately 1 km^2^) within the selected geographical area (S1 Table).

### Bioclimatic variables

The 19 bioclimatic variables from WorldClim version 2.0 (available at http://www.worldclim.org) [34] were used. Many of these variables are of great importance in limiting the distribution of species [35], for Mexico and Central America they are available at a 30" arc resolution [34], and they have demonstrated adequate performance for ENM on different species [35, 36]. A Principal Component Analysis (PCA) was conducted to assess the collinearity between bioclimatic variables [35]. The fixed cumulative eigenvalue criteria were used [37], which consists in retain the set of components that explained at least 95% of the total variance, this include the first five axes (S2 Table). The layers were re-projected using the eigenvectors values for each cell, and ASCII files of the components were created with which run the models.

Climate change scenarios were derived from three general circulation models with which the models were run, the Model for Interdisciplinary Research on Climate—Earth System Model—atmospheric Chemistry coupled version (MIROC-ESM-CHEM), The Community Climate System Model v.4 (CCSM4) and the Hadley Global Environment Model 2—Earth System (HadGEM2-ES; all available at http://www.worldclim.org) [34, 38]. Two-time horizons 2050 and 2070 were used for the three scenarios, as well as two Relative Concentration Pathways (RCP's) 2.6 and 8.5 representing an optimistic and pessimistic projection of particle concentration in the atmosphere. These models offer a Terrestrial System configuration that includes vegetation dynamics, ocean biology, and atmospheric chemistry [23], in addition providing excellent representations of climate trends for Mexico and Central America [36]. In the same way was carried out for future scenarios, preserving the first five axes of PCA. All bioclimatic layers were processed with the help of ArcMap 10.2 software [39] with SDMtoolbox functions [40].

### Potential distribution and climatic niche breadth

Ecological niches models were built using four algorithms: Gradient Boosting Machine (GBM), Support Vector Machines (SVM), Random Forest (RF), and Maximum Entropy (MaxEnt). GBMs iteratively fit regression trees to random samples, taken with replacement from a given dataset, to find optimal parameter values for predictor variables. Random forest is an algorithm that generating a set of weak learners based on a data bootstrap, the algorithm converges on an optimal solution while avoiding issues related to CARTs (Classification and Regression Trees) and parametric statistics. Breiman [41] defines Random Forest as a collection of tree-structured weak learners comprised of identically distributed random vectors where each tree contributes to a prediction for x. To the algorithms SVM and MaxEnt used background presence data, and 10,000 random background units were considered in the algorithms [42]. SVM is a set of supervised learning methods that separate presence and pseudo-absence records in a hyperplane created from the input vectors [35]. MaxEnt, to the other hand, is based on the estimation of the probable distribution of species according to a set of environmental variables, with the aim of determining the maximum distribution of entropy [42]. This algorithm achieves high predictive accuracy, in a logistic format, by improving model calibration, which provides greater representativeness of suitability [43]. GBM, Random Forest and SVM algorithms were performed in R-Studio [33] and MaxEnt was performed using MaxEnt software version 3.4 [42]. The algorithms were fed with five layers derived from the PCA to improve model prediction. The models show the potential range on a scale from 0 to 1, where 1 indicates sites of high environmental suitability for the species and 0 indicates unsuitable sites. These algorithms are integrated in the computational platform bioemsembles.

To determine the niche breadth a Jackknife analysis was performed to identify the most limiting variables for *P*. *hartwegii* [44] the seven resulting variables were analyzed through the BioClim algorithm included on modeling module of DIVA-GIS software version 7.5 [45], obtained mean, standard deviation and the intervals at which the species presents an optimal behavior. The validation of the model was performed calculating the partial receiver operating characteristic (ROC) curve [46]. This is a modification of the original ROC curve that seeks to overcome the problem of the inclusion in the area under curve (AUC) calculation of the full spectrum of proportional variables in the study area; in addition, an equal weighting of omission and commission errors [47]. Different subsets of random data were used to perform 100 replicates. In these, the occurrence data were divided into different calibration and validation groups (70%-30% respectively) to each iteration [48]. The model that presented the best performance was selected for the projection of future climate change scenarios [35, 48]. Partial ROCs were performed in R-Studio [33] with the help of the NicheToolBox library [49]. Finally, to distinguish novel environmental conditions under future climate conditions, mobility-oriented parity (MOP) analysis was used [50]. MOP identifies future environmental conditions not available in present climate conditions. Results allowed us to establish those areas of strict model extrapolation from those areas with current environmental conditions. MOP analyses was performed in NicheToolBox library [49].

### Potential distribution under different climate change scenarios

For the construction of future models, the time horizons 2050 and 2070 have been used, for the three general circulation models (MIROC-ES-CHEM, CCSM4 and HadGEM2-ES) in combination with two representative concentration pathways (RCP). The RCP 2.6 was used as an optimistic model that assumes that maximum greenhouse gas (GHG) emissions will occur between 2010 and 2020, with a consequent substantial decrease in emissions. The RCP 8.5 was used as a pessimistic model that assumes that emissions will continue to increase throughout the 21^st^ century [24]. Results of these were exported to ArcMap 10.2 [39] in order to apply a threshold value to produce the occurrence map. Applying a threshold is the last step of many species modelling approaches. It is necessary to transform the probability map in presence/absence data. Ten percentile training presence was used as suggested by Phillips and Dudík [43]. This threshold value provides a better ecologically significant result when compared with more restricted thresholds values. Finally, 12 change scenarios were presented for the suitability area of *P*. *hartwegii*. To estimate the variation in the potential distribution area, the difference between the area of the current potential distribution model and the different projection models under climate change scenarios was calculated using ArcMap 10.2 software [39].

## Results

### Potential current distribution models

**Fig 2:**
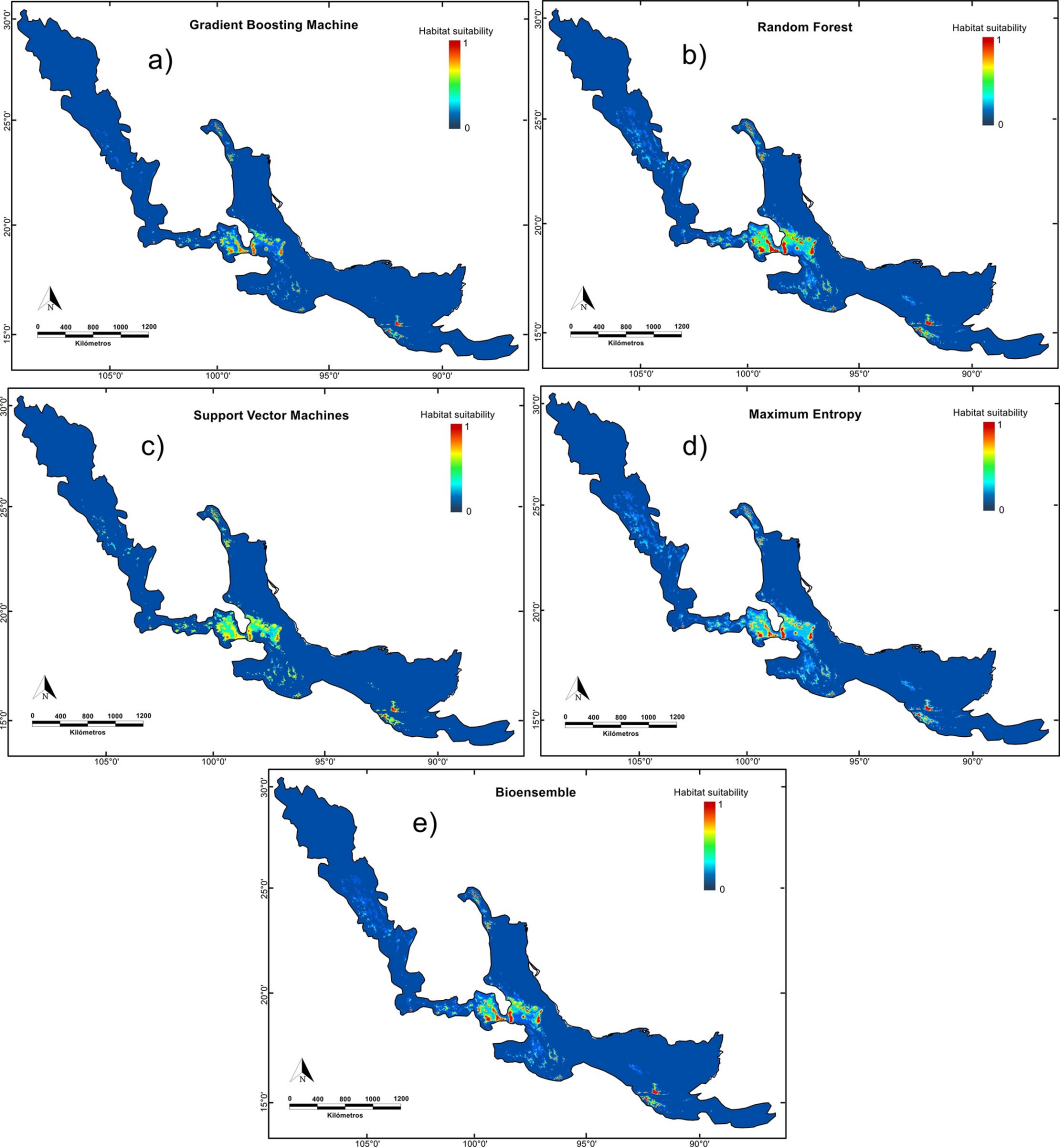
Potential current distribution models of *P*. *hartwegii* were constructed with the five main components that accumulate 95.77% of the data variation, generated by four algorithms: GBM, RF, SVM, and MaxEnt.

Potential distribution models showed a similar distribution in the four algorithms used (Fig 2). However, the estimated total area occupied by the species varied according to the algorithm used being the suitability area estimated by GBM and RF larger compared to SVM and MaxEnt. Thus, projected area was 1,903.11 km^2^ by GBM, 2,935.52 km^2^ by RF, 1,296.32 km^2^ by SVM, and 1,736.17 km^2^ by MaxEnt (Fig 2).

### Bioclimatic variables associated with *P*. *hartwegii* niche breadth

**Fig 3:**
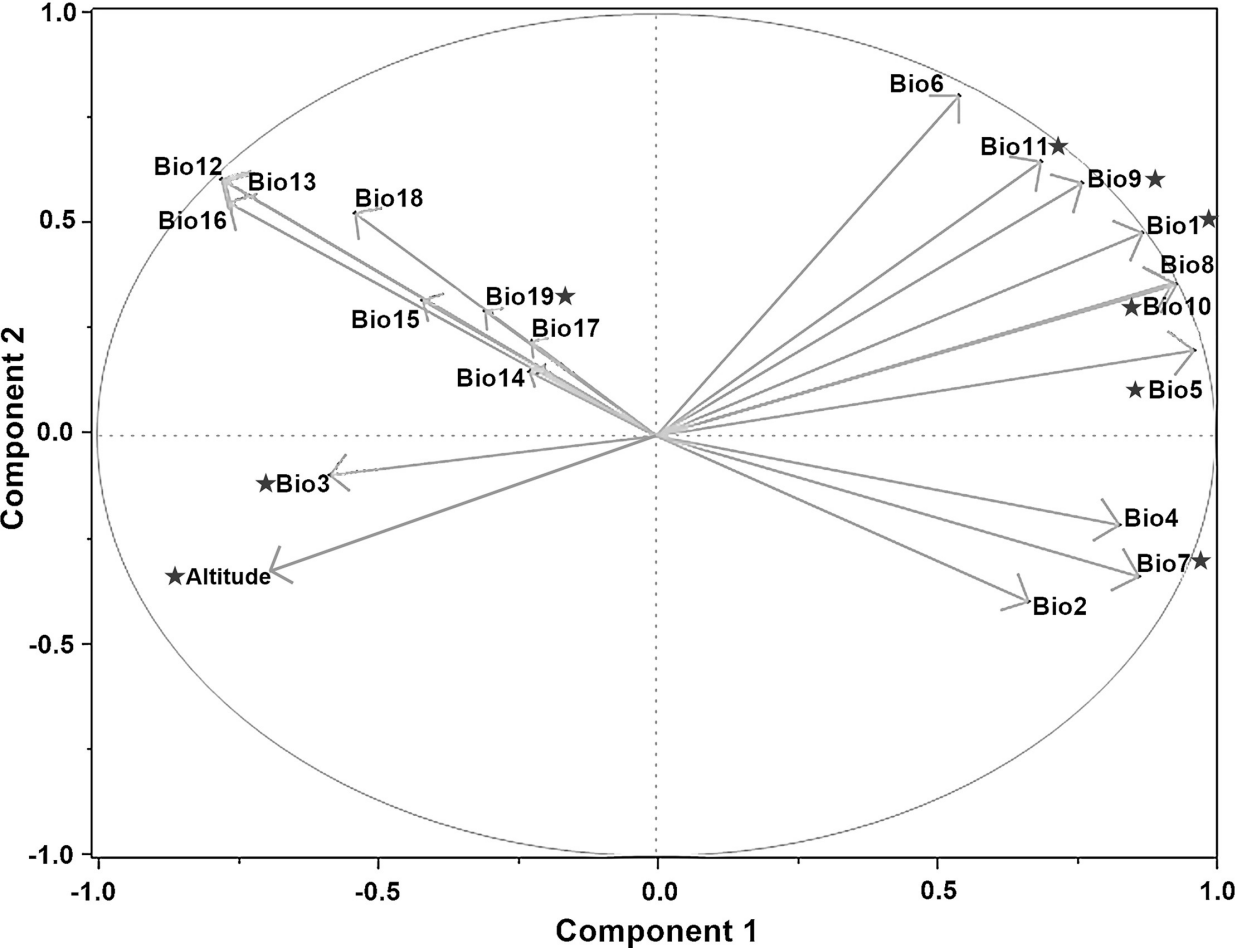
Principal components analysis of the environmental variables and altitude used in the modeling process of *P*. *hartwegii*. The symbol “★” indicates the nine variables that further limit the niche breadth.

The Jackknife test showed a greater contribution of the mean temperature of the warmest quarter (Bio10), as well as the annual mean temperature (Bio1) and the maximum temperature of the warmest month (Bio5). By omitting the precipitation of the coldest quarter (Bio19), the models gain decreases in greater proportion, which shows a greater amount of information that is not present in the other variables. PCA shows the formation of four groups of correlated variables (Fig 3) from which the biologically most important for the species were selected.

**Fig 4:**
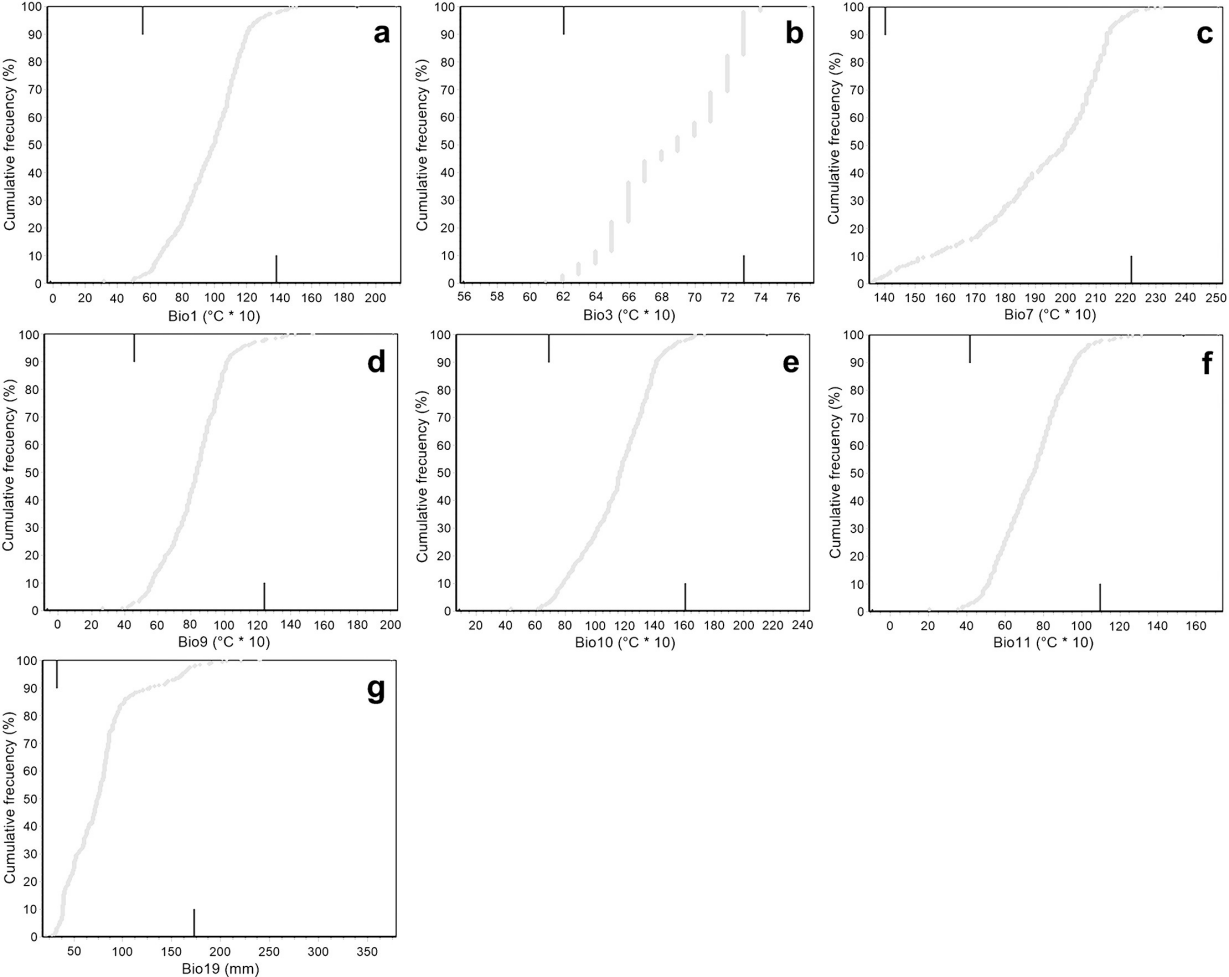
**Cumulative frequencies of the bioclimatic variables associated with the niche breadth of *P*. *hartwegii*.** Records within the intervals of each bioclimatic variables: a) mean annual temperature (Bio1); b) isothermality (Bio3); c) mean annual temperature range (Bio7); d) mean driest quarter temperature (Bio9); e) mean warmest quarter temperature (Bio10); f) mean coldest quarter temperature (Bio11); and g) coldest quarter precipitation (Bio19).

**Fig 5:**
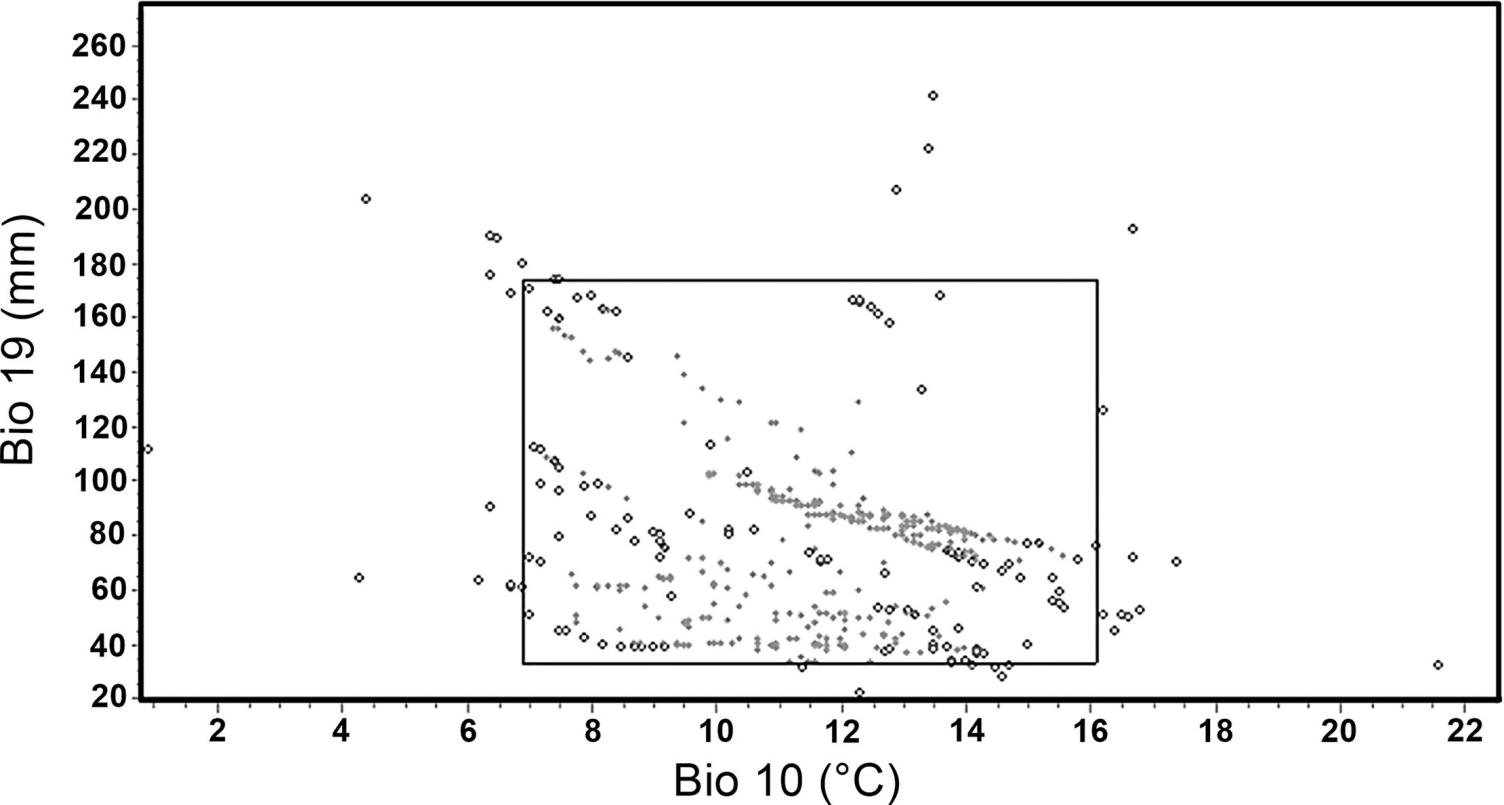
Niche breadth *of P*. *hartwegii* represented with two bioclimatic variables that provides the most information according to Jackknife analysis: The temperature of the warmest quarter (Bio10) and the precipitation of the coldest quarter (Bio19).

Narrow intervals were found for the main bioclimatic variables that determine the distribution of *P*. *hartwegii* (Fig 4), and point dispersion showed relatively low averages for all bioclimatic variables (Table 1). The temperature of the warmest quarter (Bio10) and the precipitation of the coldest quarter (Bio19) were summarized graphically, which according to the analysis were the most limiting variables for the distribution of *P*. *hartwegii*. For this reason, the niche breadth is given by the most limiting variables, Bio10 and Bio19, which provide more information in contrast to the other five variables and explaining 92.1% of the data variation (Fig 5).

**Table 1:** Bioclimatic variables and their percent contributions associated with the niche breadth of *P*. *hartwegii* according to Jackknife analysis.

Variable	Mean	Standard Error	% contribution
Bio 1	11.5°C	± 2.5°C	10.0
Bio 3	6.8°C	± 3.5°C	2.0
Bio 7	19.2°C	± 2.2°C	8.4
Bio 9	8.2°C	± 1.9°C	4.8
Bio10	11.5°C	± 2.5°C	54.5
Bio11	7.4°C	± 1.8°C	2.7
Bio19	78.9 mm	± 38.4 mm	1.2
Altitude	3192.3 m	± 18.6 m	8.4

### Models validation

The partial ROC calculations for each model showed differences in the fit of the models generated models (Table 2). GBM showed the lowest AUC value and MaxEnt the highest (Table 2). These values showed that MaxEnt was the model with the highest fit, indicating that for *P*. *hartwegii*, this model can predict the environmental suitability area of *P*. *hartwegii*.

**Table 2:** Validation by calculating the partial ROC of models generated by four algorithms used. Area under the curve (AUC) and Standard deviation (SD) values are showed for each algorithm.

Algorithm	AUC ratio	SD	AUC at 0.5	AUC ratio at 0.5
**GBM**	1.241	±0.075	0.610	0.312
**RF**	1.292	±0.063	0.615	0.319
**SVM**	1.685	±0.032	0.773	0.352
**MaxEnt**	1.787	±0.015	0.878	0.493

### Extrapolation assessment

The resulting figures of MOP analyses are available in S1 Fig, which show that there are no areas with strict extrapolation (i.e. with climate values outside the range of those in the calibration region). MOP for *P*. *hartwegii* showed a projection similarity a its calibration area. The similarity of the projection of *Pinus hartwegii* is concentrated in central Mexico, in specific, Transmexican Volcanic Belt.

### Potential distribution of *P. hartwegii* under different climate change scenarios

**Fig 6:**
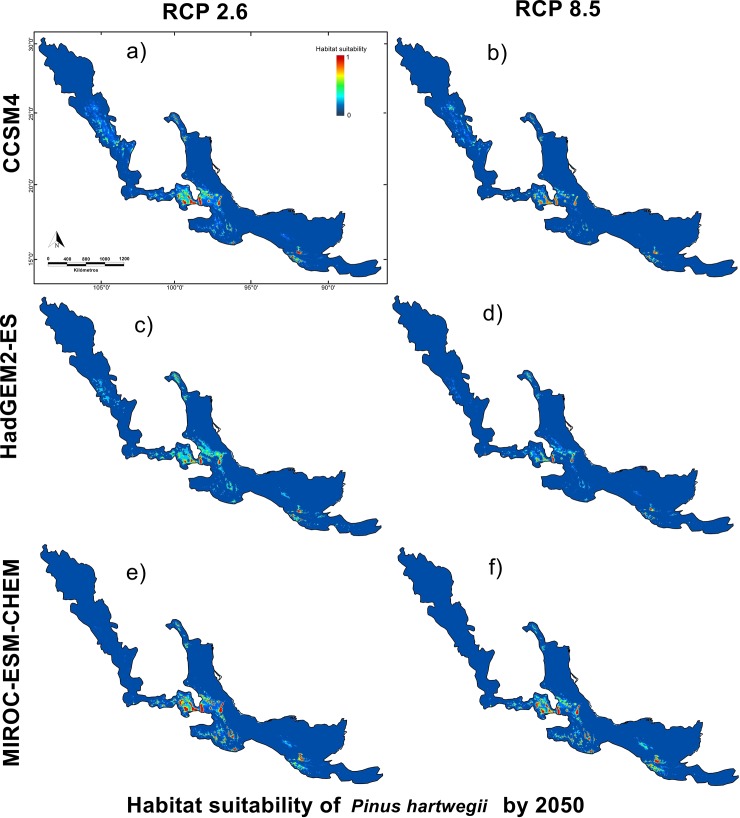
Climate niche model of *P*. *hartwegii* for 2050. Models comparing three general circulation models used MIROC-ES-CHEM, CCSM4, and HadGEM2-ES in combination with the two Relative Concentration Pathways used RCP's 2.6 and 8.5 for the horizon 2050.

**Fig 7:**
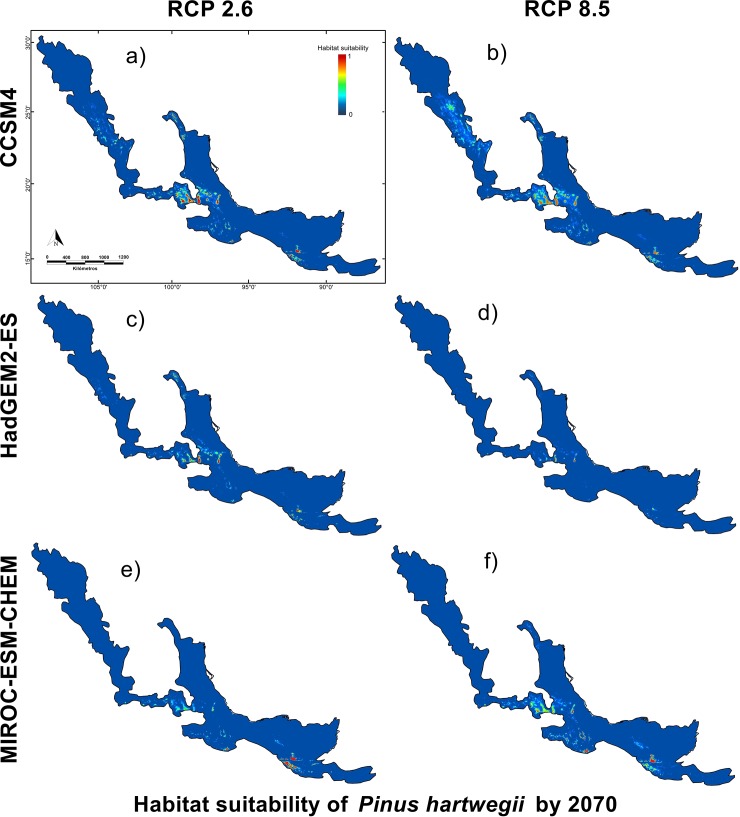
Climate niche model of *P*. *hartwegii* for 2070. Models comparing three general circulation models used MIROC-ES-CHEM, CCSM4, and HadGEM2-ES in combination with the two Relative Concentration Pathways used RCP's 2.6 and 8.5 for the horizon 2070.

The potential range of *P*. *hartwegii* distribution showed a reduction in the area occupied by the species compared to the current distribution model. All used scenarios showed a clear trend towards *P*. *hartwegii* population reduction. However, according to the results of the partial ROC curves, HadGEM2-ES was the model that best describes the species environmental suitability, showing an important reduction in the suitability area of *P*. *hartwegii* (Figs 6 and 7). HadGEM2-ES 2050–2.6 (optimistic scenario) showed a partial reduction of the area (Table 3), equivalent to 29.3% of the current distribution (Table 3, Fig 6C), compared to scenario 2050–8.5 (pessimistic scenario) which showed a reduction equivalent to 39.8% with 841.01km^2^ of residual area (Table 3, Fig 6D).

**Table 3:** Comparison between the suitability areas estimated by the current and future different general circulation models (GCM) for two Relative Concentration Pathways (RCP's) used for 2050.

GCM	Current	CCSM4		HadGEM2-ES		MIROC-ES-CHEM	
RCP		2.6	8.5	2.6	8.5	2.6	8.5
**Suitability area (Km**^**2**^**)**	1,736.17	1,593.80	1,503.52	1227.47	1,045.17	1,343.79	1,102.46
**Percentage occupied (%)**	100	91.8	86.6	70.7	60.2	77.4	63.5
**Reduced area (Km**^**2**^**)**	N/A	142.37	232.65	508.7	691	392.38	633.71
**Percentage of reduction (%)**	N/A	8.2	13.4	29.3	39.8	22.6	36.5

In contrast to the optimistic model to 2050, HadGEM2-ES-2070 showed a greater reduction in the species populations. HadGEM2-ES- 2070–2.6 showed a total reduction equivalent to 42.5% of the *P*. *hartwegii* current distribution with 803.29 km^2^ of residual area (Table 4, Fig 7C). Scenario 2070–8.5, to other hand, showed the greatest reduction in the area occupied, equivalent to 68.8% of the predicted by the current potential distribution model, occupying 435.87 km^2^ of total area (Table 4, Fig 7D).

**Table 4:** Comparison between the suitability areas estimated by the current and future different general circulation models (GCM) for two Relative Concentration Pathways (RCP's) used for 2070.

GCM	Current	CCSM4		HadGEM2-ES		MIROC-ES-CHEM	
GCM		2.6	8.5	2.6	8.5	2.6	8.5
**Suitability area (Km**^**2**^**)**	1,736.17	1,491.37	1,296.91	998.29	541.68	1,020.86	583.35
**Percentage occupied (%)**	100	85.9	74.7	57.5	31.2	58.8	33.6
**Reduced area (Km**^**2**^**)**	N/A	244.8	439.26	737.88	1,194.49	715.31	1,152.82
**Percentage of reduction (%)**	N/A	14.1	25.3	42.5	68.8	41.2	66.4

## Discussion

In this study, the potential distribution of *P*. *hartwegii* under different scenarios of climate change were determined in order to determine to what extent this species will conserve or breadth its climate niche in the face of climate change. Results showed that niche breadth of *P*. *hartwegii* has narrow ranges of suitability intervals for all bioclimatic variables evaluated, indicating that the environmental requirements of this species are very specific/restricted in their preferences [51, 52, 53]. *Pinus hartwegii* is a pine species confined to most of the highest peaks in the Mexican mountains in an altitudinal range from 3,000 to 4,200 m [29]. In these mountain areas additional to low extreme temperatures up to -30 ^o^C, other harsh condition such shallow soils, low CO_2_ partial-pressure and eventual snow presence [2, 8], increase the ecological value of this specie in the local and regional climate regulation [54] Here a strong relationship of the *P*. *hartwegii* presence with the temperature of the three months warmest of the year; in addition of precipitation of the tree coldest months of the year, i.e. winter precipitation. In areas where this species is distributed for example the Nevado de Toluca, this time period corresponds to April, May and June (warmest) and November, December and January (coldest). According to Villanueva-Díaz et al. [55] most of conifers in Mexico have a positive relationship with precipitation in the seasonal period winter-spring. This is adjudicated to role of moisture in the nutrients and carbon allocation to growth [56].

To the other hand, the fact that *P*. *hartwegii* is highly adapted to low temperatures makes also this species highly vulnerable at environmental temperature increases because of global warming [15, 20, 49, 53]. Thus, climate change predicted by the RCPs and time horizons used in this study display a greatly modify the distribution of *P*. *hartwegii* in the future. According to the results, the area occupied by this species could be reduced to 68.8% by 2070 compared to the current distribution model. This prediction is reinforced by the narrow ranges of suitability shown by the niche breadth for this species, which suggest a high specificity on the part of *P*. *hartwegii* for their population establishment. According to other projections [15, 20, 53], this species is very susceptible to being affected by climate change, due to its discontinuous distribution and the restricted range of climatic conditions to which it is adapted [49]. This, reduction in habitat suitability coincides with the results reported by other authors [14, 15, 57], who used earlier versions of the HadGEM model with projections to 2050. Gómez-Mendoza & Arriaga [14] made projections for 42 species of pines and oaks, estimating *P*. *hartwegii* as one of the species most affected by temperature increase. While Gutiérrez & Trejo [15] made projections for three species of pine and two of oak, concluding that the area occupied by *P*. *hartwegii* will be reduced up to 70% by 2050. However, these projections were made with algorithms such as GARP and BioClim which, according to several authors [33, 39, 46], tend to overestimate distribution areas. Bearing in mind that these studies have a limited time horizon and use models out to date, the projections made in the present study that show a reduction of between 29.3% and 68.8% in the distribution of *P*. *hartwegii* could be considered more realistic. These results also support the hypothesis that *P*. *hartwegii* is a highly vulnerable species to climate change however it is necessary to evaluate more closely the response of this species to temperature increase, including eco-physiological studies. *Pinus hartwegii* have been exposed to climatic variations throughout their evolutionary history [1, 15], but the pace of current changes far exceeds past patterns [49], the survival of this species to such changes will depend on its ability to adapt and on how quickly it can migrate to places with optimal climatic conditions [8, 58]. However, it is known that changes in vegetation distribution can often take hundreds or thousands of years [59]. These changes in distribution may be limited too by factors such as: availability of microsites [1, 19], the treeline shape and structure [21, 29], the health status of the vegetation [1, 60], and even the conservation status and deforestation [24].

Future distribution scenarios for *P*. *hartwegii* showed a reduction in the area of suitability associated with temperature increase at the sites where this species is found. Particularly in the populations located in the northeastern extreme in Nuevo León, Mexico, and the southern end of the distribution in El Salvador (see Fig 6), where, even for the optimistic scenario (2050–2.6), a reduction of almost 30% was observed. In addition, there are external factors such as the increment in pests and parasites related to the temperature increase [4, 58]. Aspects such as population dynamics or infestation capacity of *P*. *hartwegii* associated species such as Bark beetle (*Dendroctonus adjunctus*) or Dwarf mistletoes (*Arceuthobium globosum* and *A*. *vaginatum*) could be modified due to climate change [31, 57]. Unfortunately, there is still very little information on these pest species and how they may affect *P*. *hartwegii* populations in the future [1, 60]. On the other hand, there is an increase in anthropogenic pressure due to population growth and deforestation [31], in this regard, alternatives have been proposed such as assisted migration [19]. These alternatives which seeks to reduce decoupling between natural forest populations and the climate for which they are adapted [52], although there is still little information on the viability of these strategies on a large scale. These factors could have a negative impact on the establishment of new individuals at higher altitudes, or on the ability of trees to adapt to new conditions. In this sense, it would be favorable to evaluate in the long term not only the potential distribution of *P*. *hartwegii*, but also of the associated species and their effect on the populations of this species in relation to climate change. In addition, MOP analyses were used to minimize extrapolation errors to assess predictions [50]. In our study, the suitability areas of *P*. *hartwegii* in the models were mostly restricted to environmental zones like the calibration areas; therefore, supporting our models.

## Conclusions

The potential distribution models of *P*. *hartwegii* showed a reduction of the most suitable habitat in the populations of the latitudinal extremes of their distribution. Based on climate change projections, the suitability area will decline by between 29.3% and 68.8% over the next 50 years, which implies increased pressure on the forests made up of this species and all the biological diversity they contain. The reduction of the potential future range could be accentuated by factors such as anthropogenic pressure, driving populations from extreme northeastern and southern latitudes into extinction. Reducing the suitability area of *P*. *hartwegii* can lead to increased selection pressure in these ecosystems. The long-term effects of climate change on the populations of the species need to be assessed, as well as the genetic factors that could reduce or increase the effect of environmental conditions on these populations. Establishing long-term monitoring schemes to evaluate *in situ* the response to temperature increase of the different populations of *P*. *hartwegii* is a priority for their conservation.

## Supporting information

S1 Fig Mobility-oriented parity (MOP) analysis of Pinus hartwegii in future scenarios 2050 (A) and 2070 (B).
(PDF)

S1 Table Principal components analysis performed to extract non-collinear axes from the 20 variables used in the ecological niche modeling. (PDF)

S2 Table Occurrence data used for construction of the models of suitability area of *P*. *hartwegii*, in current and future scenarios, showing bioclimatic values for each point. (PDF)
