# Supplementary material for: Potential reduction of Hartweg´s Pine (Pinus hartwegii Lindl.) geographic distribution
Source: PLoS One. 2020 Feb 18;15(2):e0229178. doi: 10.1371/journal.pone.0229178 (PMC7028273; doi:10.1371/journal.pone.0229178)
Supplement: S1 Fig — Mobility-oriented parity (MOP) analysis of Pinus hartwegii in future scenarios 2050 (A) and 2070 (B). (PDF) [file pone.0229178.s001.pdf]

**S1 Figures Mobility-oriented parity (MOP) analysis of *Pinus hartwegii* in future scenarios 2050 (A) and 2070 (B).**

**(A)**

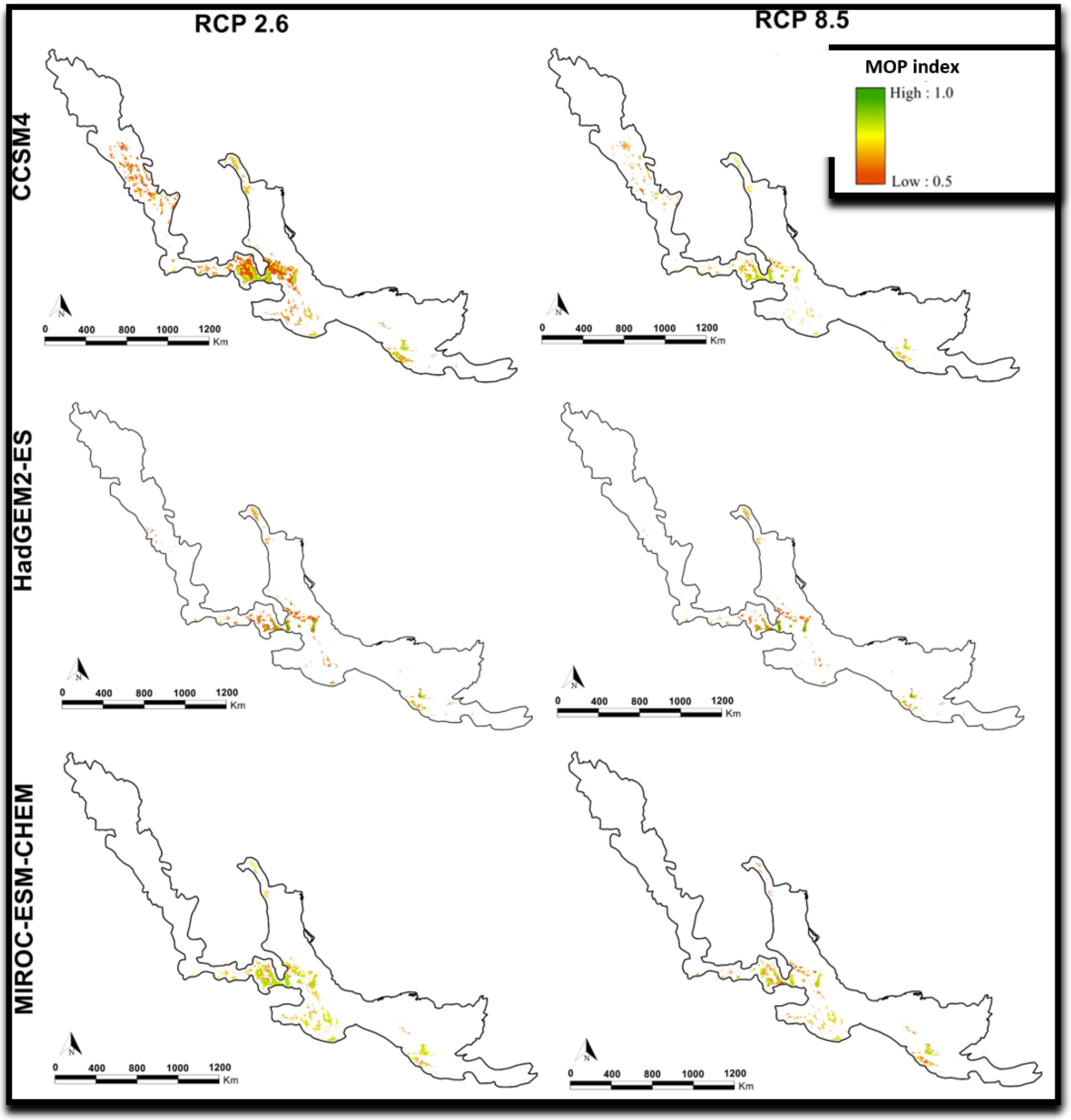

(B)

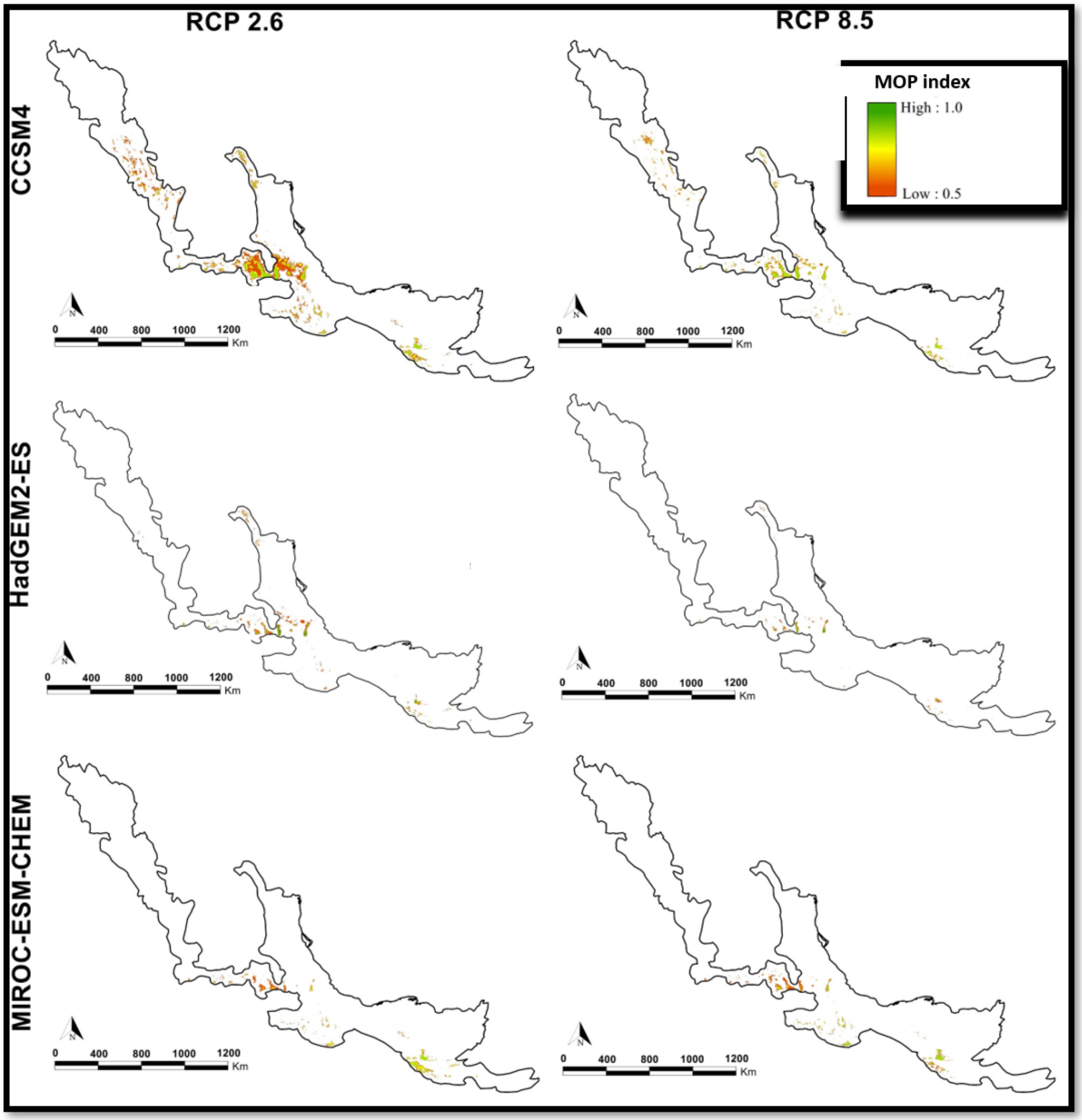
RCP 2.6
RCP 8.5
CCSM4
HadGEM2-ES
MIROC-ESM-CHEM
MOP index
High : 1.0
Low : 0.5
0 400 800 1000 1200
Km
