# Supplementary material for: Potential reduction of Hartweg´s Pine (Pinus hartwegii Lindl.) geographic distribution
Source: PLoS One. 2020 Feb 18;15(2):e0229178. doi: 10.1371/journal.pone.0229178 (PMC7028273; doi:10.1371/journal.pone.0229178)
Supplement: S1 Table — (PDF) [file pone.0229178.s002.pdf]

S1 Table Principal Components Analysis performed to extract non-collinear axes from the 20 variables used in the ecological niche modeling. Principal component axis (PC) were selected until their accumulated explanation proportion reached 95%. Loadings of PCs for each variable are being shown, as well as PC's eigenvalues, proportion of explained variance of each PC, and accumulated proportion of explained variance.

Bioclimatic Variable	PC1	PC2	PC3	PC4	PC5
Annual Mean Temperature	0.2843	0.2195	-0.0364	0.0601	-0.0078
Mean Diurnal Range	0.2043	-0.2195	-0.1124	0.4534	-0.0424
Isothermality	-0.1895	-0.0388	-0.2129	0.4489	-0.5817
Temperature Seasonality	0.2617	-0.1231	0.2241	-0.1656	0.2711
Max Temperature of Warmest Month	0.3093	0.0740	-0.0070	0.1563	0.0975
Min Temperature of Coldest Month	0.1839	0.3895	-0.0053	-0.0262	-0.0895
Temperature Annual Range	0.2698	-0.1923	-0.0053	0.2249	0.1948
Mean Temperature of Wettest Quarter	0.3005	0.1613	0.0097	-0.0228	0.0247
Mean Temperature of Driest Quarter	0.2503	0.2779	-0.0133	0.1095	0.0619
Mean Temperature of Warmest Quarter	0.3021	0.1569	0.0145	0.0341	0.0640
Mean Temperature of Coldest Quarter	0.2278	0.3050	-0.1356	0.1520	-0.1104
Annual Precipitation	-0.2401	0.3170	0.0243	0.0850	0.0617
Precipitation of Wettest Month	-0.2380	0.3156	-0.0393	0.0809	0.1404
Precipitation of Driest Month	-0.0722	0.0696	0.4965	0.2103	-0.0625
Precipitation Seasonality	-0.1316	0.1608	-0.3977	0.2471	0.3632
Precipitation of Wettest Quarter	-0.2387	0.2865	-0.0534	0.1859	0.2347
Precipitation of Driest Quarter	-0.0692	0.1072	0.5011	0.1388	-0.0948
Precipitation of Warmest Quarter	-0.1607	0.2866	-0.0769	-0.3936	-0.0077
Precipitation of Coldest Quarter	-0.0959	0.1423	0.4523	0.2507	0.0709
Altitude	-0.2019	-0.2115	0.0059	0.2418	0.5303
Principal components eigenvalue	9.765	4.0165	3.39	1.4561	0.5274
Proportion explained by each PC (%)	48.825	20.083	16.95	7.281	2.637
Accumulated proportion explained by each PC	48.825	68.908	85.858	93.138	95.775
