# Supplementary material for: Potential reduction of Hartweg´s Pine (Pinus hartwegii Lindl.) geographic distribution
Source: PLoS One. 2020 Feb 18;15(2):e0229178. doi: 10.1371/journal.pone.0229178 (PMC7028273; doi:10.1371/journal.pone.0229178)
Supplement: S2 Table — (PDF) [file pone.0229178.s003.pdf]

S2 Table Occurrence data used for construction of the models of suitability area of *P. hartwegii*, in current and future scenarios, showing bioclimatic values for each point. Data occurrence records obtained by GBIF (available in https://doi.org/10.15468/dl.g745wv).

ID	Species	Longitude	Latitude	Bio1	Bio2	Bio3	Bio4	Bio5	Bio6	Bio7	Bio8	Bio9	Bio10	Bio11	Bio12	Bio13	Bio14	Bio15	Bio16	Bio17	Bio18	Bio19	Altitude
1	Pinus_hartwegii	-103.650844	19.603345	132	128	62	1947	234	30	204	146	124	154	104	1085	216	8	93	625	27	457	56	2856
2	Pinus_hartwegii	-103.6443	19.612268	132	127	62	1944	234	30	204	145	124	154	104	1084	216	8	92	624	27	457	56	2831
3	Pinus_hartwegii	-103.63278	19.50528	134	128	63	1851	234	32	202	147	126	155	108	1114	224	8	94	651	25	464	55	2508
4	Pinus_hartwegii	-103.63111	19.47639	147	132	63	1824	248	41	207	161	138	168	121	1065	215	7	94	624	22	443	52	2140
5	Pinus_hartwegii	-103.61722	19.5125	80	112	63	1565	169	-8	177	90	73	98	58	1362	281	12	92	812	42	545	84	3401
6	Pinus_hartwegii	-103.6168	19.62153	120	124	62	1904	220	21	199	133	112	142	93	1133	229	8	93	660	29	474	61	2832
7	Pinus_hartwegii	-103.608727	19.554328	68	109	63	1501	156	-17	173	77	62	86	47	1410	292	13	91	840	47	560	93	3614
8	Pinus_hartwegii	-103.60736	19.56525	62	108	63	1445	148	-22	170	71	55	79	42	1444	300	14	90	859	51	572	98	3843
9	Pinus_hartwegii	-103.60333	19.58417	83	113	62	1644	175	-6	181	93	76	102	60	1325	274	11	92	788	41	534	82	3777
10	Pinus_hartwegii	-103.58667	19.59667	95	117	62	1755	189	1	188	106	88	115	70	1256	259	10	93	745	36	513	73	3396
11	Pinus_hartwegii	-103.58528	19.57639	98	117	61	1746	193	4	189	109	90	118	73	1249	257	10	92	741	35	511	71	3284
12	Pinus_hartwegii	-103.58222	19.54361	106	120	62	1808	203	11	192	118	98	127	81	1217	249	9	93	720	32	501	66	3037
13	Pinus_hartwegii	-103.560424	19.603583	139	132	62	1983	243	33	210	153	131	162	111	1050	211	7	94	606	24	448	51	2578
14	Pinus_hartwegii	-103.560186	19.553852	134	129	62	1931	236	30	206	148	125	156	107	1090	220	8	94	634	26	460	53	2564
15	Pinus_hartwegii	-103.552096	19.537909	145	134	63	1912	248	38	210	159	136	166	117	1050	213	7	94	611	22	445	50	2309
16	Pinus_hartwegii	-103.54167	19.575	144	134	63	1941	247	36	211	158	135	165	115	1044	211	7	93	603	24	445	51	2444
17	Pinus_hartwegii	-102.34778	19.39694	100	126	64	1593	197	3	194	109	94	118	77	1340	283	9	96	808	39	570	58	2828
18	Pinus_hartwegii	-102.34583	19.41778	91	123	65	1514	186	-2	188	100	86	108	69	1368	288	10	94	820	44	312	64	2940
19	Pinus_hartwegii	-102.34278	19.395	100	126	64	1593	197	3	194	109	94	118	77	1340	283	9	96	808	39	570	58	2886
20	Pinus_hartwegii	-102.33694	19.40889	89	122	65	1501	183	-3	186	97	84	106	67	1378	290	10	95	826	44	314	65	3362
21	Pinus_hartwegii	-102.334687	19.379671	129	139	65	1800	234	22	212	141	123	150	103	1216	261	5	99	747	25	527	40	2495
22	Pinus_hartwegii	-102.32833	19.42417	81	120	65	1426	174	-9	183	88	76	98	61	1406	294	11	93	839	48	318	71	3673
23	Pinus_hartwegii	-102.327035	19.395505	110	131	65	1650	210	10	200	120	104	128	86	1299	276	7	97	789	33	556	52	2895
24	Pinus_hartwegii	-102.32694	19.41222	80	119	65	1448	172	-9	181	88	75	96	59	1413	296	12	93	843	50	320	71	3434
25	Pinus_hartwegii	-102.32306	19.435	91	123	65	1515	186	-1	187	99	86	108	69	1366	288	10	95	820	44	311	64	3685
26	Pinus_hartwegii	-102.31972	19.45278	120	135	65	1759	222	16	206	131	113	139	94	1253	268	7	98	765	31	540	46	3338
27	Pinus_hartwegii	-102.316642	19.41668	76	118	65	1411	166	-13	179	83	71	92	55	1427	298	12	93	849	52	323	74	3494
28	Pinus_hartwegii	-102.31083	19.45889	108	131	65	1683	208	8	200	118	102	126	83	1295	276	8	97	786	36	297	53	3100
29	Pinus_hartwegii	-102.30583	19.385	102	127	65	1594	199	6	193	111	97	119	79	1340	283	9	96	808	39	306	58	2830
30	Pinus_hartwegii	-102.30444	19.42778	84	121	65	1478	177	-7	184	92	79	101	62	1396	293	11	93	834	47	317	69	3629
31	Pinus_hartwegii	-102.30194	19.39583	91	124	65	1496	186	-2	188	100	86	108	70	1377	289	10	95	827	44	313	64	3237

32	Pinus_hartwegii	-102.29278	19.44583	86	122	65	1467	179	-6	185	93	80	102	64	1386	291	11	94	829	47	316	67	3365
33	Pinus_hartwegii	-102.268267	19.41251	112	132	65	1677	213	12	201	122	106	131	88	1299	276	7	97	790	34	556	52	2970
34	Pinus_hartwegii	-102.258507	19.411719	113	132	65	1673	214	13	201	123	108	132	89	1297	276	7	97	789	34	556	51	2787
35	Pinus_hartwegii	-100.5983	19.80501	96	134	70	1318	190	1	189	102	95	111	76	1205	253	15	87	692	73	295	78	3379
36	Pinus_hartwegii	-100.583465	25.371632	109	137	63	2488	216	1	215	130	78	137	73	611	97	19	55	272	69	216	74	2567
37	Pinus_hartwegii	-100.579171	25.373263	97	135	64	2366	201	-8	209	116	83	122	62	680	110	22	54	301	76	238	84	3091
38	Pinus_hartwegii	-100.57528	25.37582	111	137	64	2515	217	3	214	133	79	139	75	599	96	18	56	268	66	213	72	3091
39	Pinus_hartwegii	-100.574327	25.365341	108	137	64	2440	213	0	213	128	77	134	72	618	99	19	55	275	69	219	75	2692
40	Pinus_hartwegii	-100.569092	25.364674	91	135	65	2309	194	-13	207	109	77	116	57	713	116	23	55	317	79	250	87	2941
41	Pinus_hartwegii	-100.568237	25.376962	110	137	64	2478	216	2	214	130	78	137	74	609	97	19	55	271	69	216	74	3455
42	Pinus_hartwegii	-100.568015	25.371097	89	134	65	2297	193	-13	206	108	76	114	56	721	117	23	54	320	80	253	90	2941
43	Pinus_hartwegii	-100.562277	25.376238	111	136	63	2481	216	3	213	132	80	138	75	606	97	19	55	270	68	214	73	3586
44	Pinus_hartwegii	-100.56028	25.36194	83	133	65	2241	185	-19	204	101	69	107	50	759	124	25	54	337	84	265	95	2747
45	Pinus_hartwegii	-100.558491	25.372855	89	134	65	2278	192	-14	206	107	75	113	55	723	118	23	54	321	80	253	90	3070
46	Pinus_hartwegii	-100.557057	25.357357	108	137	64	2417	214	1	213	128	78	135	73	619	99	19	55	275	69	220	75	2747
47	Pinus_hartwegii	-100.552	25.376	114	136	63	2503	220	6	214	135	83	141	78	586	93	18	56	262	65	208	70	3585
48	Pinus_hartwegii	-100.551418	25.357572	108	137	64	2417	214	1	213	128	78	135	73	619	99	19	55	275	69	220	75	2735
49	Pinus_hartwegii	-100.551346	25.364972	92	135	65	2303	196	-11	207	111	78	117	59	705	115	23	55	313	79	246	87	3048
50	Pinus_hartwegii	-100.549	25.369	91	134	65	2297	193	-13	206	110	77	115	57	714	117	23	55	318	79	250	87	3048
51	Pinus_hartwegii	-100.54663	25.376084	118	136	63	2532	224	9	215	140	86	146	81	567	90	17	56	255	62	203	67	3585
52	Pinus_hartwegii	-100.546005	25.355989	112	137	64	2475	218	4	214	133	81	139	76	596	95	18	55	266	66	211	72	2735
53	Pinus_hartwegii	-100.545084	25.36885	91	134	65	2297	193	-13	206	110	77	115	57	714	117	23	55	318	79	250	87	3048
54	Pinus_hartwegii	-100.544172	25.364698	84	133	65	2230	187	-17	204	102	72	109	52	748	123	24	54	332	83	261	93	2670
55	Pinus_hartwegii	-100.53849	25.367575	99	135	64	2352	204	-5	209	118	85	125	65	670	109	21	55	298	75	235	82	3039
56	Pinus_hartwegii	-100.536844	25.364793	85	133	65	2231	188	-16	204	103	72	110	53	745	122	24	54	331	82	261	92	2670
57	Pinus_hartwegii	-100.535576	25.375852	118	136	63	2565	224	9	215	141	86	146	81	565	90	17	55	254	62	201	67	3498
58	Pinus_hartwegii	-100.533143	25.357357	104	136	64	2393	208	-2	210	124	90	130	69	646	104	20	55	287	72	228	79	2739
59	Pinus_hartwegii	-100.52988	25.367575	105	136	64	2407	210	-1	211	125	75	132	70	639	104	20	55	285	72	225	77	3142
60	Pinus_hartwegii	-100.528411	25.364175	86	133	65	2210	188	-15	203	104	73	110	54	743	122	24	55	330	82	259	92	2739
61	Pinus_hartwegii	-100.528218	25.356929	94	135	65	2304	198	-9	207	113	80	119	61	701	114	22	54	310	78	245	87	2739
62	Pinus_hartwegii	-100.419214	25.220205	102	136	65	2265	205	-3	208	120	89	126	68	674	110	22	54	296	76	238	82	3362
63	Pinus_hartwegii	-100.525979	25.347989	116	137	63	2487	222	7	215	138	85	143	80	575	91	17	56	257	63	205	69	2596
64	Pinus_hartwegii	-100.393814	25.213661	92	135	65	2177	195	-10	205	109	79	115	60	730	121	24	55	321	82	255	90	3518
65	Pinus_hartwegii	-100.394217	25.199182	99	136	65	2202	203	-4	207	116	87	123	67	690	113	23	54	302	78	242	84	2912
66	Pinus_hartwegii	-100.478632	25.347646	111	136	63	2440	216	3	213	132	80	138	76	614	99	19	55	273	69	217	74	3054
67	Pinus_hartwegii	-100.520918	25.35466	91	134	65	2288	194	-12	206	110	78	116	58	714	117	23	55	317	79	250	87	3002

68	Pinus_hartwegii	-100.519102	25.361441	91	134	65	2269	194	-12	206	110	78	116	58	717	118	23	55	319	79	251	87	3002
69	Pinus_hartwegii	-100.51544	25.367855	114	136	63	2496	220	6	214	136	83	141	78	590	95	18	55	264	65	209	70	3340
70	Pinus_hartwegii	-100.515114	25.349268	105	136	64	2390	210	-1	211	125	75	131	70	643	104	20	55	285	72	227	78	3112
71	Pinus_hartwegii	-100.417906	25.21146	84	134	66	2124	186	-17	203	101	72	107	53	765	127	25	55	337	85	267	95	3137
72	Pinus_hartwegii	-100.400655	25.204084	88	134	65	2157	191	-13	204	106	76	112	57	745	123	25	54	327	83	260	92	3100
73	Pinus_hartwegii	-100.427424	25.220919	96	136	66	2197	199	-7	206	113	84	120	64	703	115	23	54	308	79	246	87	3310
74	Pinus_hartwegii	-100.512211	25.347174	105	136	64	2390	210	-1	211	125	75	131	70	643	104	20	55	285	72	227	78	2679
75	Pinus_hartwegii	-100.401488	25.211936	88	134	66	2137	190	-13	203	104	75	111	56	751	124	25	54	330	84	263	92	3464
76	Pinus_hartwegii	-100.510988	25.355758	90	134	65	2254	193	-11	204	109	77	115	57	722	118	23	54	320	80	252	90	3112
77	Pinus_hartwegii	-100.50988	25.367575	114	136	63	2496	220	6	214	136	83	141	78	590	95	18	55	264	65	209	70	3340
78	Pinus_hartwegii	-100.508769	25.360355	94	134	64	2294	198	-9	207	113	81	119	61	700	115	22	55	311	78	245	85	3112
79	Pinus_hartwegii	-100.425044	25.214792	97	136	66	2204	200	-6	206	114	85	121	65	697	114	23	54	306	79	244	86	2999
80	Pinus_hartwegii	-100.491362	25.340579	113	136	63	2458	218	4	214	134	82	140	77	601	97	18	55	268	66	213	72	2471
81	Pinus_hartwegii	-100.504329	25.366745	115	136	63	2477	220	7	213	136	84	142	79	590	95	18	55	264	65	209	70	3150
82	Pinus_hartwegii	-100.415943	25.20688	102	137	65	2286	207	-3	210	120	89	127	69	671	109	22	55	294	76	236	82	2841
83	Pinus_hartwegii	-100.485437	25.339579	109	136	64	2397	214	3	211	130	79	135	75	621	100	19	56	276	69	220	74	2528
84	Pinus_hartwegii	-100.358952	25.246199	90	133	65	2166	191	-12	203	106	78	113	58	751	125	25	55	330	84	262	92	3440
85	Pinus_hartwegii	-100.501829	25.361465	107	135	63	2358	211	0	211	126	77	133	72	635	103	20	56	283	71	224	76	3289
86	Pinus_hartwegii	-100.501751	25.354542	94	134	64	2304	198	-9	207	113	81	119	61	703	115	23	54	311	79	245	87	3289
87	Pinus_hartwegii	-100.501555	25.346408	98	135	64	2320	202	-6	208	116	84	123	64	682	112	22	55	303	76	239	84	2820
88	Pinus_hartwegii	-100.50134	25.237647	113	138	64	2396	219	5	214	134	83	139	78	599	94	19	54	263	68	213	73	2791
89	Pinus_hartwegii	-100.49531	25.360377	122	135	62	2526	228	12	216	144	91	149	86	545	86	15	56	246	58	197	64	3207
90	Pinus_hartwegii	-100.493355	25.346776	93	134	65	2276	196	-10	206	112	80	118	60	709	116	23	54	314	79	248	87	3081
91	Pinus_hartwegii	-100.490095	25.351011	127	134	62	2583	233	17	216	150	95	155	90	517	81	14	57	236	54	188	59	3207
92	Pinus_hartwegii	-100.486026	25.343825	103	135	64	2344	208	-2	210	123	90	129	69	654	107	21	55	291	73	230	79	3101
93	Pinus_hartwegii	-100.41194	25.24139	116	137	64	2348	221	8	213	136	87	142	82	598	96	19	55	264	67	212	72	2792
94	Pinus_hartwegii	-100.409612	25.214329	86	134	66	2127	188	-14	202	103	74	110	55	756	125	25	54	332	85	264	94	3340
95	Pinus_hartwegii	-100.408184	25.206833	99	136	65	2240	203	-5	208	118	87	124	67	687	112	22	54	301	77	242	84	2957
96	Pinus_hartwegii	-100.402017	25.220848	108	137	65	2294	212	2	210	127	95	133	75	644	104	21	54	282	73	228	79	3266
97	Pinus_hartwegii	-100.39191	25.24499	121	137	64	2368	226	12	214	141	91	147	87	579	92	18	55	257	64	207	69	2655
98	Pinus_hartwegii	-100.38972	25.20722	77	132	66	2059	178	-22	200	92	65	99	47	811	135	27	55	358	89	282	101	3207
99	Pinus_hartwegii	-100.386234	25.197553	92	135	66	2179	195	-9	204	109	79	116	60	732	121	24	54	321	82	256	90	2993
100	Pinus_hartwegii	-100.38111	25.20917	104	136	65	2223	208	0	208	123	92	128	72	670	110	22	55	293	76	235	82	3390
101	Pinus_hartwegii	-100.375348	25.19791	82	133	66	2105	183	-18	201	98	70	105	51	789	131	26	55	347	88	275	98	3107
102	Pinus_hartwegii	-100.373216	25.210362	107	137	65	2251	211	2	209	126	79	132	74	655	107	21	55	287	74	231	79	3388
103	Pinus_hartwegii	-100.36885	25.203812	87	133	65	2107	189	-13	202	104	76	110	56	758	126	25	54	333	85	264	94	3581

104	Pinus_hartwegii	-100.3675	25.24361	98	135	65	2209	201	-4	205	117	86	122	66	701	116	23	55	308	79	245	85	3200
105	Pinus_hartwegii	-100.366265	25.214532	107	137	65	2251	211	2	209	126	79	132	74	655	107	21	55	287	74	231	79	3223
106	Pinus_hartwegii	-100.365533	25.193711	81	132	66	2090	182	-18	200	97	69	104	51	792	133	26	54	349	88	276	98	2834
107	Pinus_hartwegii	-100.365	25.251	92	134	66	2173	194	-9	203	110	80	116	61	735	123	24	55	324	82	256	90	3200
108	Pinus_hartwegii	-100.362254	25.206904	95	135	65	2164	198	-7	205	113	83	119	64	721	120	24	54	316	81	251	88	3581
109	Pinus_hartwegii	-100.361	25.215	111	137	64	2279	215	4	211	130	82	136	78	636	104	20	55	279	71	225	76	3036
110	Pinus_hartwegii	-100.354545	25.206999	106	136	65	2243	209	0	209	124	93	130	73	668	110	22	55	293	75	235	81	3499
111	Pinus_hartwegii	-100.351077	25.2455	84	132	66	2097	184	-16	200	100	72	107	53	781	131	26	54	344	87	271	96	3422
112	Pinus_hartwegii	-100.340548	25.238242	86	132	65	2104	187	-14	201	102	73	109	55	776	131	25	55	342	86	270	95	3392
113	Pinus_hartwegii	-100.28111	19.68111	101	138	71	1274	196	3	193	107	101	115	82	1038	211	13	82	572	70	259	83	3275
114	Pinus_hartwegii	-100.275232	19.671445	101	138	71	1302	196	2	194	107	100	115	82	1040	212	13	82	573	70	259	83	3308
115	Pinus_hartwegii	-100.275	19.67917	101	138	71	1274	196	3	193	107	101	115	82	1038	211	13	82	572	70	259	83	3275
116	Pinus_hartwegii	-100.261322	24.870235	116	143	66	2191	223	8	215	134	89	140	85	640	105	22	54	272	75	227	78	2727
117	Pinus_hartwegii	-100.261138	24.855847	127	146	66	2270	234	15	219	146	99	152	94	604	97	22	52	252	73	213	77	2553
118	Pinus_hartwegii	-100.258787	24.880338	112	142	66	2186	218	5	213	130	85	136	80	659	108	23	54	281	77	233	81	3063
119	Pinus_hartwegii	-100.252268	24.863253	112	142	66	2167	218	5	213	129	86	136	80	662	109	23	54	282	77	234	81	2761
120	Pinus_hartwegii	-100.25	24.833	135	149	67	2316	243	21	222	154	108	161	102	581	92	22	51	238	72	204	76	2346
121	Pinus_hartwegii	-100.249936	24.877673	101	139	66	2083	206	-2	208	117	90	124	71	707	118	24	55	304	79	249	87	3063
122	Pinus_hartwegii	-100.249936	24.887762	108	141	66	2116	213	2	211	124	97	131	77	677	112	23	54	289	77	239	83	3112
123	Pinus_hartwegii	-100.249841	24.872533	109	141	67	2108	213	3	210	125	98	131	78	677	112	23	54	289	77	239	83	2938
124	Pinus_hartwegii	-100.24944	19.575	104	139	71	1309	199	4	195	111	104	119	85	1033	209	12	81	567	67	255	85	3073
125	Pinus_hartwegii	-100.248687	24.901027	117	143	66	2183	223	9	214	135	91	142	86	644	106	22	54	274	75	227	79	2926
126	Pinus_hartwegii	-100.248175	24.892283	108	141	67	2116	212	2	210	124	96	131	77	683	114	23	55	293	78	240	84	3147
127	Pinus_hartwegii	-100.244041	24.873706	94	138	67	2056	198	-7	205	110	84	117	64	740	124	25	54	319	82	260	91	3180
128	Pinus_hartwegii	-100.243083	24.878815	89	136	67	1992	191	-11	202	104	78	111	60	767	129	26	55	332	85	269	94	3286
129	Pinus_hartwegii	-100.242626	24.843994	114	143	66	2184	220	6	214	131	87	139	82	656	108	23	54	278	77	231	81	2560
130	Pinus_hartwegii	-100.242155	24.856115	109	142	66	2143	214	2	212	126	97	132	77	677	112	23	54	289	77	239	83	2810
131	Pinus_hartwegii	-100.241084	24.885001	102	139	67	2067	206	-1	207	118	91	125	72	707	118	24	55	304	80	249	86	3330
132	Pinus_hartwegii	-100.240787	24.894626	110	141	66	2141	215	4	211	127	99	134	79	674	112	23	54	288	77	238	82	3152
133	Pinus_hartwegii	-100.240335	24.84066	112	142	66	2133	217	5	212	129	101	135	81	669	111	23	54	284	77	236	82	2734
134	Pinus_hartwegii	-100.24018	24.864205	85	135	67	1990	186	-15	201	99	73	106	55	793	133	27	54	343	88	278	98	3302
135	Pinus_hartwegii	-100.239835	24.904834	115	142	66	2171	220	7	213	132	88	138	83	659	109	23	54	281	77	232	81	2885
136	Pinus_hartwegii	-100.237503	24.897196	110	141	66	2141	215	4	211	127	99	134	79	674	112	23	54	288	77	238	82	3152
137	Pinus_hartwegii	-100.236915	24.843458	102	140	67	2068	206	-2	208	118	92	125	72	709	118	24	54	303	80	249	87	2734
138	Pinus_hartwegii	-100.236261	24.871206	78	133	66	1965	180	-20	200	92	68	100	49	826	139	28	55	360	91	289	102	3494
139	Pinus_hartwegii	-100.236218	24.90136	115	142	66	2171	220	7	213	132	88	138	83	659	109	23	54	281	77	232	81	2885

140	Pinus_hartwegii	-100.23514	24.87787	84	135	67	1972	186	-14	200	98	73	106	55	796	134	27	54	345	88	279	98	3596
141	Pinus_hartwegii	-100.234065	24.855401	94	137	66	2057	197	-8	205	109	83	116	64	745	125	25	54	322	82	262	91	3129
142	Pinus_hartwegii	-100.233761	24.864266	85	135	67	1990	186	-15	201	99	73	106	55	793	133	27	54	343	88	278	98	3302
143	Pinus_hartwegii	-100.233363	24.887726	100	139	67	2060	205	-2	207	116	89	123	70	716	120	24	55	308	81	252	88	3499
144	Pinus_hartwegii	-100.232442	24.834702	115	143	66	2174	221	7	214	132	88	140	84	657	109	23	54	279	77	232	81	2734
145	Pinus_hartwegii	-100.22947	24.89243	108	140	66	2133	213	3	210	125	98	132	77	685	114	23	54	293	78	242	84	3114
146	Pinus_hartwegii	-100.229306	24.857423	93	137	66	2043	197	-8	205	108	83	116	64	750	126	25	54	323	84	264	92	3147
147	Pinus_hartwegii	-100.227712	24.8485	101	140	67	2059	205	-2	207	116	91	124	71	713	120	24	54	306	80	251	87	2626
148	Pinus_hartwegii	-100.22764	24.86135	82	134	67	1985	183	-17	200	96	71	104	52	811	137	27	54	352	89	284	101	3309
149	Pinus_hartwegii	-100.227197	24.837412	115	143	66	2174	221	7	214	132	88	140	84	657	109	23	54	279	77	232	81	2626
150	Pinus_hartwegii	-100.2257	24.86784	78	133	66	1941	179	-20	199	92	67	99	49	828	140	28	54	361	91	290	102	3567
151	Pinus_hartwegii	-100.225368	24.904501	116	142	66	2202	222	8	214	134	89	140	84	653	108	23	54	278	77	230	80	2924
152	Pinus_hartwegii	-100.22397	24.89007	104	140	67	2069	208	0	208	121	94	127	74	703	118	24	54	301	80	247	86	3429
153	Pinus_hartwegii	-100.221513	24.849107	119	144	66	2178	225	10	215	136	92	143	87	645	107	22	54	274	75	228	78	3431
154	Pinus_hartwegii	-100.221228	24.892699	114	142	66	2151	219	7	212	132	87	138	83	664	111	23	54	283	77	234	81	3001
155	Pinus_hartwegii	-100.221049	24.856876	111	142	66	2103	216	4	212	128	101	134	80	674	113	23	54	288	77	238	82	2743
156	Pinus_hartwegii	-100.22015	24.884266	104	140	67	2069	208	0	208	121	94	127	74	703	118	24	54	301	80	247	86	3288
157	Pinus_hartwegii	-100.22005	24.872462	97	138	67	2046	200	-5	205	113	86	120	67	733	124	25	54	316	82	257	90	3127
158	Pinus_hartwegii	-100.219621	24.863824	101	139	67	2057	204	-2	206	117	90	123	71	718	121	24	55	308	81	252	88	2956
159	Pinus_hartwegii	-100.219546	19.628216	108	141	71	1334	204	6	198	115	107	122	88	980	196	12	81	534	65	240	86	3192
160	Pinus_hartwegii	-100.21333	24.88833	116	142	66	2167	221	8	213	133	89	139	84	661	110	23	54	281	77	233	81	2999
161	Pinus_hartwegii	-100.212721	24.857304	125	145	66	2222	232	15	217	143	98	150	93	626	103	22	53	263	73	221	77	3477
162	Pinus_hartwegii	-100.211769	24.862254	116	143	66	2175	222	8	214	134	89	140	85	660	110	23	54	280	77	233	81	2678
163	Pinus_hartwegii	-100.211722	24.870796	109	141	66	2110	213	2	211	126	98	132	78	688	115	24	54	293	79	242	84	2830
164	Pinus_hartwegii	-100.211	24.882	108	141	67	2113	213	3	210	125	82	132	77	692	116	24	54	296	80	243	84	2908
165	Pinus_hartwegii	-100.205035	24.881718	122	144	66	2198	228	13	215	141	95	147	91	639	106	22	54	270	75	225	78	2908
166	Pinus_hartwegii	-99.867303	23.762175	108	142	67	2042	214	3	211	121	101	130	78	773	142	20	61	336	76	288	83	3055
167	Pinus_hartwegii	-99.861	23.816	115	144	67	2079	222	8	214	129	108	138	84	753	140	20	62	327	74	280	80	3114
168	Pinus_hartwegii	-99.860967	23.815981	115	144	67	2079	222	8	214	129	108	138	84	753	140	20	62	327	74	280	80	3114
169	Pinus_hartwegii	-99.859957	23.769878	87	135	67	1842	188	-11	199	98	81	107	59	873	159	24	60	385	86	319	98	3241
170	Pinus_hartwegii	-99.851	23.801	96	138	67	1916	199	-4	203	108	89	117	68	833	153	23	60	365	83	305	92	3378
171	Pinus_hartwegii	-99.850742	23.794298	88	135	68	1844	190	-8	198	100	82	109	61	865	159	24	60	382	85	316	96	3378
172	Pinus_hartwegii	-99.84472	23.80233	91	136	67	1898	194	-7	201	102	84	112	63	860	158	24	60	379	84	314	96	3348
173	Pinus_hartwegii	-99.838355	19.115738	101	133	71	1230	192	5	187	106	90	116	84	1065	200	16	80	568	71	306	74	3377
174	Pinus_hartwegii	-99.825	19.20833	110	137	70	1352	204	9	195	116	98	127	92	953	184	14	80	503	66	275	69	3208
175	Pinus_hartwegii	-99.817539	23.88708	110	142	67	2069	216	5	211	124	103	133	80	795	150	21	62	346	79	292	84	3107

176	Pinus_hartwegii	-99.817182	23.895004	108	141	67	2058	214	4	210	122	101	131	78	798	150	21	62	348	79	293	84	3091
177	Pinus_hartwegii	-99.816944	23.880656	106	140	67	1994	210	3	207	119	99	128	76	810	152	22	61	354	80	296	87	2898
178	Pinus_hartwegii	-99.809	23.879	114	143	67	2085	220	8	212	129	90	138	84	784	149	21	62	342	78	288	82	2898
179	Pinus_hartwegii	-99.80833	19.15833	95	132	70	1182	186	0	186	99	84	109	78	1075	208	16	82	582	67	307	69	3429
180	Pinus_hartwegii	-99.807356	23.883478	122	145	67	2155	230	14	216	138	99	147	91	764	147	20	64	332	76	282	78	3011
181	Pinus_hartwegii	-99.80361	19.12333	84	128	71	1086	173	-6	179	87	74	98	69	1150	227	18	84	632	66	329	66	3660
182	Pinus_hartwegii	-99.80139	19.14639	88	130	71	1143	178	-3	181	92	78	102	72	1120	220	17	83	613	66	320	67	3547
183	Pinus_hartwegii	-99.800288	23.902785	121	145	67	2162	228	13	215	137	97	145	89	771	148	20	63	335	76	284	78	2865
184	Pinus_hartwegii	-99.799	23.896	131	148	66	2258	240	19	221	148	106	156	98	749	148	19	65	327	72	277	72	2865
185	Pinus_hartwegii	-99.796806	23.902087	129	147	66	2232	238	17	221	146	104	154	97	753	148	19	65	329	73	278	74	2865
186	Pinus_hartwegii	-99.79094	19.13946	80	127	71	1079	168	-9	177	83	71	93	65	1157	232	18	85	639	64	331	64	3708
187	Pinus_hartwegii	-99.77667	19.10583	57	120	72	920	141	-24	165	61	44	69	44	1263	265	19	86	709	61	366	61	4072
188	Pinus_hartwegii	-99.775	19.14167	80	128	72	1069	168	-9	177	83	71	93	65	1152	231	18	84	637	63	330	63	3955
189	Pinus_hartwegii	-99.77417	19.12833	65	123	72	969	151	-18	169	70	52	78	52	1212	250	19	85	674	61	352	61	4134
190	Pinus_hartwegii	-99.76222	19.11333	55	120	72	933	140	-25	165	60	43	67	43	1263	266	19	86	710	61	366	61	3000
191	Pinus_hartwegii	-99.75833	19.74167	85	134	71	1244	178	-9	187	89	68	100	68	983	197	17	82	533	61	272	61	3613
192	Pinus_hartwegii	-99.741198	23.686443	132	147	66	2305	242	20	222	149	107	158	99	749	146	17	65	328	70	284	71	2619
193	Pinus_hartwegii	-99.740555	23.682088	118	143	67	2128	225	12	213	132	94	141	87	797	154	20	64	351	77	298	80	2723
194	Pinus_hartwegii	-99.738917	20.864413	189	169	67	2496	311	60	251	210	154	216	154	439	83	6	74	216	27	155	32	1798
195	Pinus_hartwegii	-99.737634	23.690426	124	144	66	2218	232	15	217	139	99	148	91	780	151	19	64	343	74	293	77	2817
196	Pinus_hartwegii	-99.731524	23.682367	117	142	66	2110	223	11	212	131	93	140	86	809	156	20	64	357	77	302	81	2856
197	Pinus_hartwegii	-99.73028	19.11944	72	126	72	1021	159	-14	173	75	58	85	58	1185	241	18	86	658	59	344	59	3913
198	Pinus_hartwegii	-99.728301	23.686942	115	141	66	2122	222	9	213	129	91	139	84	814	157	20	64	360	78	304	82	3005
199	Pinus_hartwegii	-99.728111	23.70298	101	138	67	1964	205	0	205	114	95	123	73	862	164	22	63	382	83	318	90	3182
200	Pinus_hartwegii	-99.728111	23.681279	117	142	66	2110	223	11	212	131	93	140	86	809	156	20	64	357	77	302	81	2925
201	Pinus_hartwegii	-99.727339	23.670504	108	139	66	2054	213	4	209	121	101	130	78	839	161	21	64	373	80	311	86	3089
202	Pinus_hartwegii	-99.723637	23.665575	105	138	66	2009	209	3	206	118	99	127	75	855	163	22	63	379	82	316	89	2971
203	Pinus_hartwegii	-99.718193	23.672647	112	140	66	2076	217	8	209	126	88	135	81	830	160	21	63	367	80	308	84	3053
204	Pinus_hartwegii	-99.718	23.663	105	138	66	2009	209	3	206	118	99	127	75	855	163	22	63	379	82	316	89	3116
205	Pinus_hartwegii	-99.717732	23.656328	107	139	66	2059	212	3	209	120	100	130	77	846	162	21	64	376	80	314	87	3068
206	Pinus_hartwegii	-99.717404	23.677708	119	142	66	2190	226	12	214	135	95	144	87	803	156	19	65	354	76	300	79	2861
207	Pinus_hartwegii	-99.716547	23.6473	113	140	66	2096	218	7	211	127	89	136	82	823	159	20	64	365	78	307	83	2840
208	Pinus_hartwegii	-99.711803	23.652928	113	141	67	2087	219	9	210	127	90	137	83	826	160	20	64	366	79	308	83	3068
209	Pinus_hartwegii	-99.711503	23.672758	109	139	66	2051	214	5	209	123	103	132	79	842	162	21	63	373	80	312	86	3053
210	Pinus_hartwegii	-99.711243	23.630989	120	142	66	2187	227	13	214	135	95	144	88	804	156	19	64	356	76	302	79	2713
211	Pinus_hartwegii	-99.711	23.631	120	142	66	2187	227	13	214	135	95	144	88	804	156	19	64	356	76	302	79	2833

212	Pinus_hartwegii	-99.710712	23.663157	110	140	66	2020	215	6	209	123	104	132	80	842	162	21	63	373	80	312	86	3033
213	Pinus_hartwegii	-99.7087	19.16464	104	136	70	1300	197	5	192	110	86	120	86	1025	193	16	81	553	64	297	66	3306
214	Pinus_hartwegii	-99.705	23.611	127	144	66	2240	235	18	217	143	102	152	95	783	153	18	65	346	73	296	76	2597
215	Pinus_hartwegii	-99.704853	23.61099	127	144	66	2240	235	18	217	143	102	152	95	783	153	18	65	346	73	296	76	2597
216	Pinus_hartwegii	-99.70428	21.155	125	139	65	2059	233	22	211	136	97	149	96	960	208	14	82	507	56	250	70	2600
217	Pinus_hartwegii	-99.7005	21.16112	117	137	66	1977	224	17	207	128	102	140	90	967	207	15	81	508	59	253	73	2949
218	Pinus_hartwegii	-99.7005	21.16112	117	137	66	1977	224	17	207	128	102	140	90	967	207	15	81	508	59	253	73	2949
219	Pinus_hartwegii	-99.69972	21.15944	117	137	66	1977	224	17	207	128	102	140	90	967	207	15	81	508	59	253	73	2600
220	Pinus_hartwegii	-99.68906	21.15726	118	137	66	1991	224	17	207	129	102	141	91	971	209	15	82	511	59	253	73	2930
221	Pinus_hartwegii	-99.6675	21.14944	141	144	65	2240	253	33	220	154	111	167	110	1005	216	15	82	529	59	259	72	2716
222	Pinus_hartwegii	-99.5975	20.83383	120	139	66	1988	226	17	209	130	94	143	92	874	183	12	83	462	47	237	60	2928
223	Pinus_hartwegii	-99.56444	20.91528	149	149	66	2239	262	38	224	162	119	174	118	1062	217	17	80	553	62	270	70	2422
224	Pinus_hartwegii	-99.507412	19.589964	89	136	70	1252	184	-8	192	93	72	104	72	1011	200	14	84	558	52	287	53	3580
225	Pinus_hartwegii	-99.5	19.56056	84	135	71	1247	178	-11	189	89	68	100	67	1028	204	15	84	566	53	294	54	3491
226	Pinus_hartwegii	-99.46472	19.51194	101	141	71	1361	198	0	198	106	83	117	82	1046	205	13	87	588	49	294	51	3407
227	Pinus_hartwegii	-99.426995	19.479526	93	138	71	1321	189	-5	194	98	76	110	75	1053	207	13	87	590	48	299	51	3462
228	Pinus_hartwegii	-99.424949	19.486141	95	139	70	1341	192	-4	196	100	78	112	77	1055	207	13	87	593	48	298	51	3519
229	Pinus_hartwegii	-99.40833	19.30833	111	145	70	1448	210	4	206	118	92	129	92	1225	253	11	93	713	46	344	53	3150
230	Pinus_hartwegii	-99.39111	19.305	119	148	69	1520	220	8	212	130	99	137	98	1208	249	11	94	714	47	355	55	3047
231	Pinus_hartwegii	-99.39028	19.41667	114	147	70	1526	214	4	210	120	93	132	92	1173	240	11	92	677	45	331	51	3212
232	Pinus_hartwegii	-99.380669	19.441177	106	144	70	1425	205	0	205	112	87	123	87	1126	226	11	91	649	44	314	49	3208
233	Pinus_hartwegii	-99.375	19.41333	113	147	70	1483	214	4	210	119	93	131	93	1180	242	11	92	683	44	332	51	3175
234	Pinus_hartwegii	-99.375	19.425	102	142	70	1387	200	-2	202	107	83	119	83	1109	220	12	90	636	45	310	49	3166
235	Pinus_hartwegii	-99.37	19.41	116	148	69	1547	218	5	213	126	95	135	94	1189	244	12	92	691	44	340	52	3175
236	Pinus_hartwegii	-99.302092	19.277702	95	139	71	1270	190	-4	194	100	78	110	77	1144	226	11	90	655	42	327	48	3440
237	Pinus_hartwegii	-99.292714	19.270562	107	145	70	1396	207	1	206	113	89	125	88	1255	262	10	94	742	41	343	49	3172
238	Pinus_hartwegii	-99.269434	19.270504	116	150	70	1529	217	4	213	127	95	134	95	1270	266	10	96	754	41	363	50	3113
239	Pinus_hartwegii	-99.26353	19.22344	96	140	71	1272	192	-3	195	101	79	113	79	1178	235	11	91	679	41	336	41	3692
240	Pinus_hartwegii	-99.258034	19.204836	78	133	72	1099	169	-14	183	84	63	91	63	1111	222	13	88	622	41	331	49	3495
241	Pinus_hartwegii	-99.25671	19.21205	82	134	71	1140	175	-12	187	85	67	96	66	1114	222	12	89	626	40	330	48	3816
242	Pinus_hartwegii	-99.25111	19.22139	101	142	71	1296	197	-2	199	107	83	116	83	1207	245	10	93	703	39	340	47	3662
243	Pinus_hartwegii	-99.248996	19.256186	124	155	69	1601	229	7	222	132	102	143	102	1127	233	9	95	664	36	317	36	2995
244	Pinus_hartwegii	-99.22667	19.24389	121	153	69	1575	225	6	219	133	99	139	99	1168	240	9	95	688	37	333	37	2957
245	Pinus_hartwegii	-99.225	19.225	121	153	69	1578	225	6	219	133	99	140	99	1185	245	9	96	701	38	337	38	3086
246	Pinus_hartwegii	-99.20833	19.125	111	147	71	1421	210	3	207	121	91	128	91	1306	271	10	96	771	40	369	47	3021
247	Pinus_hartwegii	-99.195	19.11333	118	150	70	1522	218	5	213	129	96	135	96	1332	278	9	97	799	40	376	40	3036

248	Pinus_hartwegii	-99.193949	19.121314	117	150	70	1499	218	6	212	128	96	135	96	1321	276	9	97	791	39	374	45	3037
249	Pinus_hartwegii	-99.16667	19.13333	119	152	70	1538	221	5	216	130	97	137	97	1296	270	9	97	777	39	368	39	3092
250	Pinus_hartwegii	-99.1425	19.08833	103	144	72	1306	199	-1	200	109	85	118	85	1255	254	10	94	730	37	362	37	3307
251	Pinus_hartwegii	-99.141845	19.095198	106	145	71	1338	203	0	203	115	87	121	87	1275	260	10	94	744	38	366	38	3307
252	Pinus_hartwegii	-99.135183	19.087584	99	142	71	1280	195	-3	198	104	81	115	81	1230	246	10	93	710	37	357	46	3306
253	Pinus_hartwegii	-99.083	19.131	118	153	70	1531	221	4	217	129	97	136	97	1255	258	9	96	746	37	363	42	3042
254	Pinus_hartwegii	-99.017875	19.055461	114	151	71	1455	215	4	211	124	94	131	94	1283	261	9	96	760	36	375	36	3058
255	Pinus_hartwegii	-98.742928	19.512199	121	165	71	1773	231	-1	232	135	96	140	96	704	127	8	79	370	30	236	34	2968
256	Pinus_hartwegii	-98.738083	19.218517	120	161	72	1551	226	3	223	132	98	137	98	950	180	9	87	533	34	307	39	2722
257	Pinus_hartwegii	-98.726321	19.331193	102	152	73	1376	203	-5	208	111	83	118	82	988	183	10	86	547	32	319	39	3196
258	Pinus_hartwegii	-98.725749	19.32158	106	154	72	1420	208	-4	212	116	86	121	85	979	181	10	86	542	34	318	39	3137
259	Pinus_hartwegii	-98.725	19.34167	92	146	73	1315	190	-10	200	100	74	107	73	987	184	9	86	545	30	316	40	3284
260	Pinus_hartwegii	-98.721697	20.192215	118	128	67	1568	214	23	191	122	99	138	97	920	163	18	70	436	58	279	77	3005
261	Pinus_hartwegii	-98.720654	19.331184	104	153	72	1441	205	-5	210	114	84	120	83	982	181	10	87	543	33	319	39	3196
262	Pinus_hartwegii	-98.720654	19.331184	104	153	72	1441	205	-5	210	114	84	120	83	982	181	10	87	543	33	319	39	3196
263	Pinus_hartwegii	-98.71889	19.37	65	132	73	1066	156	-24	180	70	51	78	50	1003	192	9	85	545	32	318	47	3894
264	Pinus_hartwegii	-98.7175	19.335	101	152	73	1402	202	-6	208	111	81	117	81	982	182	10	87	544	32	317	39	3212
265	Pinus_hartwegii	-98.71194	19.53056	122	165	71	1745	232	0	232	136	97	141	97	675	123	8	78	352	29	230	32	2917
266	Pinus_hartwegii	-98.7102	19.130184	122	159	71	1522	227	6	221	132	99	138	99	1069	205	9	90	608	34	334	34	2796
267	Pinus_hartwegii	-98.709129	19.139107	117	158	72	1494	221	3	218	128	95	133	95	1079	204	10	89	609	36	342	36	2853
268	Pinus_hartwegii	-98.70583	19.25194	86	142	73	1197	182	-12	194	93	70	100	69	994	188	8	88	552	27	320	40	3230
269	Pinus_hartwegii	-98.70537	19.41128	71	135	72	1138	164	-21	185	77	56	85	55	979	185	9	85	531	31	312	45	3976
270	Pinus_hartwegii	-98.70105	19.571923	119	162	71	1721	227	-1	228	132	94	138	94	722	133	8	79	382	30	244	33	2973
271	Pinus_hartwegii	-98.69389	19.03972	102	149	73	1284	202	-2	204	110	84	117	84	1148	219	10	91	653	31	362	42	3216
272	Pinus_hartwegii	-98.69296	19.528855	120	164	71	1730	229	-1	230	133	95	138	95	712	132	8	79	375	30	242	33	2962
273	Pinus_hartwegii	-98.69167	19.24167	86	142	72	1224	182	-13	195	93	69	100	68	993	188	8	87	551	27	321	40	3529
274	Pinus_hartwegii	-98.69167	19.35833	95	147	72	1326	193	-9	202	103	76	110	76	979	181	10	86	540	31	316	40	3228
275	Pinus_hartwegii	-98.69167	19.375	89	144	73	1257	186	-11	197	97	72	104	71	974	181	9	85	535	31	313	41	3409
276	Pinus_hartwegii	-98.69167	19.375	89	144	73	1257	186	-11	197	97	72	104	71	974	181	9	85	535	31	313	41	3409
277	Pinus_hartwegii	-98.69151	19.07989	115	156	72	1405	217	3	214	124	95	130	94	1145	218	10	91	651	34	360	43	2968
278	Pinus_hartwegii	-98.680057	20.180318	115	129	67	1557	211	20	191	119	96	135	94	918	162	18	70	436	57	278	76	2965
279	Pinus_hartwegii	-98.67682	19.09095	98	148	73	1257	196	-5	201	106	80	112	80	1099	209	10	90	621	30	350	41	3194
280	Pinus_hartwegii	-98.675619	19.346969	110	159	72	1515	214	-4	218	121	89	127	88	944	176	10	85	517	34	315	37	3058
281	Pinus_hartwegii	-98.671582	19.103982	86	142	73	1163	181	-12	193	93	70	99	69	1028	197	8	89	576	26	332	40	3347
282	Pinus_hartwegii	-98.67028	19.29583	101	152	73	1386	202	-6	208	110	82	117	81	1003	186	10	87	556	32	326	39	3290
283	Pinus_hartwegii	-98.66972	19.3525	113	161	73	1504	216	-3	219	124	91	128	91	928	173	10	84	505	35	310	38	2995

284	Pinus_hartwegii	-98.66667	19.1275	89	144	73	1202	186	-10	196	96	73	103	72	1038	198	8	89	582	27	334	40	3888
285	Pinus_hartwegii	-98.66667	19.15	69	134	73	1070	161	-22	183	74	55	82	54	960	187	6	87	532	23	314	40	3831
286	Pinus_hartwegii	-98.66389	19.36667	109	158	72	1503	213	-5	218	120	88	126	87	936	174	10	84	512	34	311	38	3006
287	Pinus_hartwegii	-98.65833	19.09167	79	139	73	1116	173	-16	189	85	63	92	63	987	191	7	88	551	24	321	40	3673
288	Pinus_hartwegii	-98.65361	19.07333	74	136	73	1075	166	-18	184	79	59	87	59	979	190	6	88	545	22	321	39	3734
289	Pinus_hartwegii	-98.65	19.09361	78	139	73	1117	173	-16	189	84	63	92	63	983	190	6	89	549	23	321	39	3689
290	Pinus_hartwegii	-98.64618	19.14466	58	130	73	998	148	-29	177	62	45	70	44	1064	207	8	86	583	32	337	51	4017
291	Pinus_hartwegii	-98.646002	19.043901	67	133	73	1010	158	-22	180	71	53	79	53	1005	197	6	89	559	24	326	42	4002
292	Pinus_hartwegii	-98.64333	19.10444	75	137	74	1094	168	-17	185	81	60	88	60	967	187	6	88	538	23	317	39	3728
293	Pinus_hartwegii	-98.64278	19.27917	101	152	73	1370	201	-6	207	110	82	116	81	1011	187	10	87	561	32	329	40	3240
294	Pinus_hartwegii	-98.64167	19.05833	69	134	73	1052	161	-21	182	74	55	82	55	980	192	6	89	546	23	320	40	3992
295	Pinus_hartwegii	-98.64167	19.05833	69	134	73	1052	161	-21	182	74	55	82	55	980	192	6	89	546	23	320	40	3992
296	Pinus_hartwegii	-98.64167	19.05833	69	134	73	1052	161	-21	182	74	55	82	55	980	192	6	89	546	23	320	40	3992
297	Pinus_hartwegii	-98.64167	19.09167	80	139	73	1124	174	-15	189	86	65	94	64	993	191	7	88	554	24	324	40	3651
298	Pinus_hartwegii	-98.586147	19.088069	105	152	73	1337	205	-2	207	114	86	120	86	1116	210	10	89	628	31	359	41	3181
299	Pinus_hartwegii	-98.63397	19.26888	100	151	72	1377	200	-7	207	109	81	116	80	1014	188	10	87	563	31	330	39	3428
300	Pinus_hartwegii	-98.63097	20.13972	118	129	67	1569	214	22	192	122	98	138	97	874	152	18	69	410	57	269	74	2867
301	Pinus_hartwegii	-98.62738	19.02056	-1	108	74	705	76	-69	145	-3	-6	9	-9	1757	336	30	78	934	104	475	111	4468
302	Pinus_hartwegii	-98.6267	19.06775	77	138	73	1085	171	-16	187	83	62	90	62	987	191	6	88	551	23	323	39	3886
303	Pinus_hartwegii	-98.61607	19.12851	72	136	73	1080	164	-20	184	78	57	85	57	958	185	6	88	531	22	315	39	3809
304	Pinus_hartwegii	-98.614177	19.029921	32	119	73	837	116	-45	161	32	27	43	21	1389	270	17	83	748	64	405	64	4597
305	Pinus_hartwegii	-98.57778	19.1375	104	153	73	1324	205	-3	208	112	85	118	85	1087	203	10	89	609	31	352	41	3144
306	Pinus_hartwegii	-98.558855	19.037357	112	155	72	1395	214	1	213	122	92	127	92	1163	220	10	91	655	32	369	43	3060
307	Pinus_hartwegii	-98.552651	19.220677	128	163	71	1652	236	9	227	141	104	146	104	920	175	8	88	508	28	300	28	2762
308	Pinus_hartwegii	-98.29167	19.98583	142	153	68	1796	252	29	223	155	118	164	118	613	112	11	73	295	34	199	45	2440
309	Pinus_hartwegii	-98.09	19.23111	111	156	72	1489	216	2	214	121	90	127	89	986	182	10	83	527	33	336	42	2954
310	Pinus_hartwegii	-98.075921	19.263971	113	157	72	1495	219	3	216	123	92	129	92	962	178	10	83	512	32	330	40	3045
311	Pinus_hartwegii	-98.068069	19.20615	106	154	73	1408	210	0	210	115	86	121	85	1016	186	11	84	543	35	342	45	3094
312	Pinus_hartwegii	-98.067355	19.272537	112	157	73	1504	218	3	215	122	91	129	90	962	177	10	82	511	33	329	41	3068
313	Pinus_hartwegii	-98.04167	19.27889	108	155	73	1444	213	1	212	118	88	124	87	974	178	11	82	516	35	332	43	3173
314	Pinus_hartwegii	-98.03167	19.23	51	128	73	1011	141	-33	174	53	45	64	38	1344	250	18	81	713	63	192	90	3974
315	Pinus_hartwegii	-98.025953	19.296808	118	159	72	1596	225	6	219	129	96	135	95	909	167	9	82	477	31	317	38	3018
316	Pinus_hartwegii	-98.02304	19.22647	62	132	73	1093	154	-26	180	67	55	75	47	1262	233	16	82	668	57	377	79	3975
317	Pinus_hartwegii	-97.967418	19.279675	125	160	72	1630	233	12	221	136	102	142	101	859	154	8	81	440	30	306	36	2889
318	Pinus_hartwegii	-97.458973	19.305115	107	148	72	1386	208	5	203	112	88	123	87	977	177	17	80	512	51	300	59	3015
319	Pinus_hartwegii	-97.325	19.09167	91	141	73	1216	188	-3	191	94	84	105	73	1452	282	26	84	807	81	180	103	3229

320	Pinus_hartwegii	-97.31667	19.08333	85	139	73	1196	182	-7	189	88	79	99	68	1538	299	28	84	855	86	184	113	3313
321	Pinus_hartwegii	-97.31666	19.08333	85	139	73	1196	182	-7	189	88	79	99	68	1538	299	28	84	855	86	184	113	3313
322	Pinus_hartwegii	-97.29167	19.09167	64	129	72	1073	155	-22	177	65	58	77	49	1797	341	34	81	978	105	208	152	3743
323	Pinus_hartwegii	-97.28333	19.08333	70	132	73	1090	163	-17	180	72	64	83	55	1750	335	33	82	959	102	202	144	3751
324	Pinus_hartwegii	-97.28333	19.08333	70	132	73	1090	163	-17	180	72	64	83	55	1750	335	33	82	959	102	202	144	3751
325	Pinus_hartwegii	-97.26039	19.481639	126	141	70	1664	224	25	199	135	103	145	103	475	92	9	72	220	30	166	31	2579
326	Pinus_hartwegii	-97.25833	19.075	60	128	73	1046	150	-24	174	62	54	73	46	1866	354	36	81	1014	111	213	162	3991
327	Pinus_hartwegii	-97.25833	19.49167	128	140	70	1747	226	26	200	138	104	147	104	460	93	9	72	213	30	158	32	2533
328	Pinus_hartwegii	-97.25278	19.04722	32	117	73	895	116	-43	159	32	27	44	21	2091	386	43	77	1107	133	242	203	4441
329	Pinus_hartwegii	-97.25	19.13333	88	139	73	1210	185	-4	189	92	81	102	71	1556	307	29	85	874	90	401	115	3525
330	Pinus_hartwegii	-97.245942	19.485237	124	139	70	1692	220	22	198	132	100	142	100	530	102	11	70	248	37	178	38	2572
331	Pinus_hartwegii	-97.243543	19.478727	124	140	71	1653	220	23	197	132	101	142	100	530	101	10	71	249	36	178	37	2613
332	Pinus_hartwegii	-97.23333	19.04167	58	127	74	992	147	-24	171	60	53	70	45	1915	363	38	80	1038	116	216	170	4045
333	Pinus_hartwegii	-97.215733	19.088406	98	142	72	1294	197	2	195	101	91	113	79	1475	297	28	86	836	85	378	108	3480
334	Pinus_hartwegii	-97.209546	19.068501	81	136	73	1165	175	-9	184	84	74	95	65	1719	337	33	83	958	101	432	138	3604
335	Pinus_hartwegii	-97.21167	19.09278	104	144	73	1338	203	6	197	108	96	119	84	1362	275	25	85	771	79	354	98	3367
336	Pinus_hartwegii	-97.193425	19.130046	112	146	73	1345	212	13	199	117	105	127	93	1206	243	23	85	680	71	317	85	2768
337	Pinus_hartwegii	-97.19167	19.50833	99	134	71	1437	194	6	188	105	89	116	79	1325	263	29	83	736	92	341	103	3372
338	Pinus_hartwegii	-97.18861	19.53444	100	133	71	1452	194	7	187	106	90	117	80	1258	249	29	82	686	91	327	102	3041
339	Pinus_hartwegii	-97.184873	19.556288	106	134	70	1522	201	12	189	113	95	124	85	1025	196	25	76	528	79	279	93	2954
340	Pinus_hartwegii	-97.184205	19.079483	104	144	73	1341	203	6	197	108	96	119	84	1398	284	26	86	795	81	357	103	3138
341	Pinus_hartwegii	-97.175	19.50833	83	129	71	1286	175	-5	180	87	75	98	65	1609	320	35	82	894	110	396	133	3647
342	Pinus_hartwegii	-97.16404	19.46663	64	124	72	1136	152	-19	171	67	57	78	49	1816	346	39	79	984	122	442	167	3719
343	Pinus_hartwegii	-97.16333	19.49667	60	122	72	1111	148	-21	169	62	54	74	45	1842	350	40	78	992	125	447	174	3969
344	Pinus_hartwegii	-97.1625	19.47889	56	121	72	1085	142	-24	166	57	49	69	41	1883	354	41	78	1009	128	457	180	3774
345	Pinus_hartwegii	-97.159518	19.105954	117	146	73	1352	217	18	199	122	110	132	97	1169	236	23	84	658	70	303	86	2893
346	Pinus_hartwegii	-97.15833	19.50833	67	124	72	1166	156	-16	172	70	60	82	52	1777	342	39	79	964	122	432	163	3773
347	Pinus_hartwegii	-97.15833	19.50833	67	124	72	1166	156	-16	172	70	60	82	52	1777	342	39	79	964	122	432	163	3773
348	Pinus_hartwegii	-97.15833	19.525	86	129	72	1296	177	-2	179	90	78	101	68	1587	319	35	83	887	110	389	129	3643
349	Pinus_hartwegii	-97.152619	19.460633	68	125	71	1164	158	-16	174	71	61	82	52	1793	344	38	80	979	121	435	162	3488
350	Pinus_hartwegii	-97.150595	19.096436	118	146	73	1379	219	19	200	123	111	134	98	1165	235	23	84	655	70	301	87	2662
351	Pinus_hartwegii	-97.14944	19.48639	50	119	72	1074	136	-28	164	52	44	64	36	1929	361	42	77	1028	132	466	190	4055
352	Pinus_hartwegii	-97.148479	19.470913	61	123	72	1125	149	-20	169	64	55	75	46	1855	352	40	79	1002	125	450	174	3743
353	Pinus_hartwegii	-97.1475	19.49306	52	120	72	1059	137	-28	165	54	45	65	38	1924	361	42	77	1026	132	465	189	3990
354	Pinus_hartwegii	-97.142911	19.462204	69	125	72	1177	158	-15	173	72	62	83	53	1796	345	38	80	982	121	435	162	3470
355	Pinus_hartwegii	-97.141055	19.511459	69	125	72	1195	158	-14	172	72	62	84	53	1777	344	39	80	968	122	430	162	3977

356	Pinus_hartwegii	-97.137343	19.455637	89	131	71	1318	182	-1	183	93	80	104	70	1648	329	35	85	940	110	400	128	3143
357	Pinus_hartwegii	-97.133916	19.488616	79	128	72	1258	170	-7	177	83	71	94	62	1706	336	37	82	948	116	414	145	3393
358	Pinus_hartwegii	-97.133203	19.498039	67	124	72	1148	155	-16	171	70	59	80	51	1813	348	39	79	985	124	438	168	3666
359	Pinus_hartwegii	-97.13	19.48	93	132	71	1380	187	2	185	98	84	109	74	1622	328	35	87	936	110	393	121	3137
360	Pinus_hartwegii	-97.128063	19.47077	93	132	71	1374	187	2	185	98	85	110	74	1626	328	35	87	939	110	394	121	3258
361	Pinus_hartwegii	-97.125624	19.58032	105	131	70	1507	197	12	185	111	94	122	83	1101	213	28	75	564	89	288	110	2928
362	Pinus_hartwegii	-97.120354	19.489044	98	132	71	1395	191	6	185	103	88	114	78	1568	318	34	87	905	107	383	118	3123
363	Pinus_hartwegii	-96.65333	17.19306	110	130	73	961	203	25	178	112	106	123	98	1647	303	24	78	835	87	204	128	2699
364	Pinus_hartwegii	-96.55833	17.54167	146	134	69	1319	243	51	192	148	139	162	127	1368	256	16	83	712	54	345	126	2988
365	Pinus_hartwegii	-96.53333	17.18333	140	133	72	1107	234	50	184	142	135	154	125	1122	233	9	88	614	46	131	64	2643
366	Pinus_hartwegii	-96.525	17.61694	120	130	70	1176	215	31	184	123	115	135	104	2258	394	33	79	1166	121	187	241	2888
367	Pinus_hartwegii	-96.50278	17.51667	120	129	71	1103	214	33	181	123	115	134	105	2183	388	33	79	1126	119	194	222	2727
368	Pinus_hartwegii	-96.45278	17.38167	115	127	72	996	206	31	175	117	111	129	102	2103	372	34	77	1063	122	213	207	2641
369	Pinus_hartwegii	-96.43694	17.385	122	128	71	1097	213	35	178	124	117	136	107	1877	347	25	81	975	99	184	168	3033
370	Pinus_hartwegii	-96.41667	17.58333	151	127	69	1349	243	60	183	154	143	167	132	1741	344	22	81	925	76	380	192	1823
371	Pinus_hartwegii	-96.28076	16.20801	88	113	77	530	163	18	145	85	83	96	82	1631	305	19	82	832	70	233	88	3587
372	Pinus_hartwegii	-96.25889	16.18	83	112	77	553	158	14	144	80	78	92	78	1689	316	21	82	862	75	239	75	3545
373	Pinus_hartwegii	-96.001818	17.0279	121	115	73	882	202	45	157	122	117	133	110	1663	299	25	81	861	92	182	133	2336
374	Pinus_hartwegii	-92.109845	15.136015	69	110	71	881	139	-14	153	76	56	77	56	1805	346	16	79	862	65	755	65	3000
375	Pinus_hartwegii	-92.109813	15.119525	87	115	72	962	159	0	159	94	72	96	72	1669	322	11	82	796	46	712	46	2886
376	Pinus_hartwegii	-92.107782	15.132075	63	109	72	836	133	-18	151	69	51	72	51	1858	355	17	79	888	70	774	70	3000
377	Pinus_hartwegii	-92.100861	15.137709	63	108	71	818	132	-19	151	68	50	70	50	1865	356	17	78	891	72	775	72	3020
378	Pinus_hartwegii	-92.092445	15.118822	97	118	71	1030	171	7	164	105	82	107	82	1596	307	9	83	759	37	688	37	3150
379	Pinus_hartwegii	-92.058893	15.196917	105	120	72	1019	180	14	166	113	90	115	90	1499	284	8	83	704	33	648	33	3011
380	Pinus_hartwegii	-91.908653	15.041488	79	112	71	961	151	-5	156	87	65	89	65	1716	334	13	81	816	53	723	53	3426
381	Pinus_hartwegii	-91.607901	15.656758	110	118	73	697	187	27	160	115	100	117	100	1371	249	18	70	594	70	463	70	3302
382	Pinus_hartwegii	-91.601238	15.656163	109	117	73	719	186	26	160	114	99	117	99	1384	250	18	70	600	71	465	71	3000
383	Pinus_hartwegii	-91.552047	15.514503	67	107	72	790	137	-11	148	72	57	75	56	1780	317	25	70	799	101	707	105	3000
384	Pinus_hartwegii	-91.551336	14.756021	84	110	69	1072	156	-2	158	92	68	95	68	1724	354	12	84	835	47	728	47	3120
385	Pinus_hartwegii	-91.547462	15.523338	63	107	72	793	133	-15	148	68	53	71	51	1828	325	27	70	822	107	723	112	2999
386	Pinus_hartwegii	-91.544909	15.513313	67	107	71	790	137	-12	149	72	57	74	55	1789	318	26	70	802	103	710	107	3000
387	Pinus_hartwegii	-91.543957	15.506175	66	107	71	796	137	-13	150	71	56	74	54	1796	320	26	70	806	103	561	107	3504
388	Pinus_hartwegii	-91.542667	15.521077	63	107	72	793	133	-15	148	68	53	71	51	1828	325	27	70	822	107	723	112	3023
389	Pinus_hartwegii	-91.541913	15.517567	63	107	72	793	133	-15	148	68	53	71	51	1828	325	27	70	822	107	723	112	2809
390	Pinus_hartwegii	-91.540914	15.529421	64	107	72	788	134	-14	148	69	54	72	52	1815	322	27	70	815	106	718	111	3000
391	Pinus_hartwegii	-91.53389	15.49694	65	107	71	795	136	-13	149	70	55	73	53	1803	322	26	70	810	103	715	108	3045

392	Pinus_hartwegii	-91.533012	15.528066	74	109	72	792	145	-6	151	79	64	81	62	1712	304	24	71	763	97	681	99	3812
393	Pinus_hartwegii	-91.528757	15.52094	71	108	71	808	143	-8	151	77	61	79	59	1741	309	25	70	777	99	548	102	3223
394	Pinus_hartwegii	-91.52083	15.52444	76	109	72	795	147	-4	151	81	65	83	64	1697	301	24	71	755	95	676	97	3420
395	Pinus_hartwegii	-91.52	14.8	109	117	68	1333	185	13	172	119	90	123	90	1416	308	7	88	698	22	610	22	3600
396	Pinus_hartwegii	-91.48222	15.53194	94	113	72	784	168	12	156	99	82	102	82	1513	265	20	71	658	80	496	80	3002
397	Pinus_hartwegii	-91.48056	15.57778	98	114	73	771	172	16	156	104	87	106	87	1492	261	21	70	645	82	490	82	2880
398	Pinus_hartwegii	-91.395274	14.889367	103	114	68	1176	178	12	166	111	85	114	85	1418	299	9	85	684	31	600	31	3000
399	Pinus_hartwegii	-91.349826	14.843444	101	113	68	1156	175	11	164	110	84	112	84	1455	308	9	86	706	33	616	33	3650
400	Pinus_hartwegii	-91.327459	14.912567	96	111	69	1095	168	9	159	104	80	107	80	1502	308	11	83	716	41	631	41	3500
401	Pinus_hartwegii	-91.317465	14.913281	92	110	69	1070	164	6	158	99	76	102	76	1567	317	12	82	744	48	655	48	3220
402	Pinus_hartwegii	-91.301523	14.913757	100	112	69	1100	173	12	161	108	83	110	83	1455	301	10	84	696	38	612	38	3810
403	Pinus_hartwegii	-91.191449	14.580442	104	108	68	1166	175	17	158	113	89	116	87	1603	352	9	89	805	33	684	33	3000
404	Pinus_hartwegii	-91.190117	14.586057	89	105	68	1066	158	5	153	97	75	99	73	1755	368	12	85	862	47	740	49	3615
405	Pinus_hartwegii	-91.188064	14.585873	89	105	68	1066	158	5	153	97	75	99	73	1755	368	12	85	862	47	740	49	2870
406	Pinus_hartwegii	-91.185599	14.5825	83	104	68	1070	152	1	151	90	69	93	67	1821	376	14	84	889	54	764	57	2990
407	Pinus_hartwegii	-91.179647	14.581679	102	107	68	1176	173	16	157	110	87	114	84	1625	354	9	88	813	35	692	35	3010
408	Pinus_hartwegii	-90.882325	14.497941	74	98	69	962	140	-1	141	80	62	84	60	2019	401	18	80	974	73	838	82	3140
409	Pinus_hartwegii	-90.879774	14.505847	63	96	69	920	127	-11	138	69	51	72	49	2139	417	22	78	1024	88	877	99	3120
410	Pinus_hartwegii	-90.879529	14.489739	81	99	69	999	147	5	142	88	69	91	66	1949	392	16	82	947	64	816	72	3120
411	Pinus_hartwegii	-90.877259	14.47399	77	98	69	984	142	1	141	83	64	87	62	2004	401	17	81	972	69	835	78	3000
412	Pinus_hartwegii	-90.87576	14.488197	81	99	69	999	147	5	142	88	69	91	66	1949	392	16	82	947	64	816	72	2990
413	Pinus_hartwegii	-90.873782	14.482198	103	101	68	1100	171	24	147	110	90	114	86	1759	367	12	86	870	44	574	50	2900
414	Pinus_hartwegii	-90.872821	14.505802	66	97	69	937	131	-8	139	72	53	75	52	2111	413	21	79	1012	84	868	96	3000
415	Pinus_hartwegii	-90.870349	14.518022	95	100	68	1050	163	17	146	103	82	105	79	1800	369	13	84	882	51	583	57	3252
416	Pinus_hartwegii	-90.869993	14.498591	71	97	69	956	136	-4	140	77	59	80	56	2056	406	19	79	990	77	851	87	3260
417	Pinus_hartwegii	-90.868823	14.471779	92	100	69	1011	159	15	144	98	79	102	77	1860	381	14	83	912	54	599	60	3650
418	Pinus_hartwegii	-90.748243	14.465938	76	97	69	967	141	2	139	83	64	86	62	2049	404	19	80	988	75	852	86	3000
419	Pinus_hartwegii	-90.748005	14.470756	82	97	68	971	148	7	141	88	70	91	67	1995	395	18	80	964	70	630	80	3500
420	Pinus_hartwegii	-90.742948	14.475812	111	99	68	1063	179	35	144	118	98	121	95	1741	357	11	86	860	42	571	48	2900
421	Pinus_hartwegii	-90.742541	14.467602	82	97	68	971	148	7	141	88	70	91	67	1995	395	18	80	964	70	630	80	3760
422	Pinus_hartwegii	-90.741376	14.464492	76	97	69	967	141	2	139	83	64	86	62	2049	404	19	80	988	75	852	86	3610
423	Pinus_hartwegii	-90.741342	14.457491	106	99	68	1045	174	30	144	113	94	116	90	1788	366	12	85	881	46	583	53	3804
424	Pinus_hartwegii	-90.73638	14.465	80	97	69	996	145	6	139	87	67	90	65	2013	398	18	81	972	71	841	81	4006
425	Pinus_hartwegii	-90.736107	14.457491	107	98	69	1040	174	32	142	115	95	118	92	1781	364	12	85	878	45	582	51	3902
426	Pinus_hartwegii	-90.734977	14.476229	110	99	68	1036	178	34	144	117	98	120	95	1749	357	12	86	861	44	574	51	4079
427	Pinus_hartwegii	-90.73472	14.4725	82	97	69	971	148	8	140	89	70	91	68	1988	394	17	81	962	68	629	78	3797

428	Pinus_hartwegii	-90.730278	14.466057	110	98	69	1050	177	35	142	117	99	121	95	1760	360	12	86	868	44	576	51	3809
429	Pinus_hartwegii	-90.730278	14.470934	99	98	69	1019	166	24	142	106	87	109	84	1842	372	14	84	901	53	595	61	3648
430	Pinus_hartwegii	-88.700387	14.53803	111	91	66	1322	176	40	136	119	97	123	90	2234	381	33	67	976	135	669	165	3000
431	Pinus_hartwegii	-88.692979	14.547694	111	91	66	1342	177	40	137	119	98	123	90	2228	379	33	67	972	135	667	166	3000
432	Pinus_hartwegii	-88.69282	14.528037	116	91	66	1325	182	45	137	124	103	128	95	2194	376	31	68	962	128	661	158	3100
433	Pinus_hartwegii	-88.69282	14.53803	110	91	66	1341	175	39	136	118	96	122	88	2235	381	33	67	976	135	669	166	3202
434	Pinus_hartwegii	-88.68333	14.53333	114	91	66	1341	180	43	137	122	101	126	93	2206	377	32	68	965	131	663	161	3720
435	Pinus_hartwegii	-88.68333	14.54166	113	91	66	1343	179	42	137	121	100	125	92	2210	377	33	67	965	133	663	164	3000
436	Pinus_hartwegii	-99.77814	19.08781	70	124	72	987	157	-15	172	73	57	83	57	1212	247	18	85	674	61	351	61	3949
437	Pinus_hartwegii	-99.7515	19.12856	68	124	72	1000	155	-17	172	71	55	81	55	1199	246	18	86	667	60	347	60	4080
438	Pinus_hartwegii	-99.77365	19.11962	55	120	73	920	138	-26	164	59	42	67	42	1267	266	20	86	710	62	366	62	3000
439	Pinus_hartwegii	-99.77421	19.11347	51	119	73	887	134	-28	162	54	39	62	39	1293	271	20	86	723	63	372	63	3000
440	Pinus_hartwegii	-99.75517	19.13556	72	126	72	996	159	-14	173	75	59	85	59	1183	240	18	85	655	61	343	61	4080
441	Pinus_hartwegii	-98.57663	19.213585	109	156	72	1428	211	-3	214	119	88	125	88	1052	196	10	88	585	33	343	33	3147
442	Pinus_hartwegii	-99.79027	19.09307	73	125	72	1002	160	-12	172	75	59	86	59	1198	243	18	86	665	61	346	61	3689
443	Pinus_hartwegii	-99.78776	19.11393	68	124	72	985	155	-17	172	71	55	81	55	1210	248	19	86	672	61	350	61	3804
444	Pinus_hartwegii	-99.7594	19.14312	79	128	72	1073	167	-10	177	82	64	92	64	1157	232	18	85	639	63	333	63	4006
445	Pinus_hartwegii	-99.7302	19.1256	76	127	72	1026	163	-12	175	79	62	89	62	1168	236	17	85	648	60	338	60	3902
446	Pinus_hartwegii	-98.60997	19.16226	63	132	74	1014	153	-25	178	67	49	75	49	1008	196	7	87	555	27	325	45	4079
447	Pinus_hartwegii	-98.61498	19.14367	73	137	73	1114	167	-19	186	79	58	87	58	958	185	6	88	531	22	315	39	3797
448	Pinus_hartwegii	-98.62356	19.12651	72	136	73	1080	164	-20	184	78	57	85	57	958	185	6	88	531	22	315	39	3809
449	Pinus_hartwegii	-98.66936	19.15583	69	134	73	1070	161	-22	183	74	55	82	54	960	187	6	87	532	23	314	40	3648
450	Pinus_hartwegii	-98.6758	19.19372	74	137	73	1109	168	-18	186	80	59	88	59	959	185	7	87	530	24	313	40	3779
451	Pinus_hartwegii	-98.69011	19.22233	81	140	73	1153	176	-15	191	87	65	95	65	978	187	7	87	543	25	318	39	3535
452	Pinus_hartwegii	-98.67365	19.26022	87	143	73	1232	183	-12	195	94	70	101	69	992	187	8	87	550	27	321	40	3637
453	Pinus_hartwegii	-98.72303	19.41824	64	132	73	1103	155	-25	180	70	50	78	49	1011	193	10	84	547	34	318	50	3875
454	Pinus_hartwegii	-99.76048	19.7377	76	131	71	1217	168	-15	183	79	60	91	60	1026	206	18	81	557	63	286	64	3613
455	Pinus_hartwegii	-99.76831	19.74552	87	134	71	1247	180	-7	187	91	70	102	70	972	196	17	81	528	60	268	60	3545
456	Pinus_hartwegii	-99.33896	19.11429	85	134	72	1116	176	-9	185	89	70	98	69	1161	232	12	90	658	42	341	49	3531
457	Pinus_hartwegii	-99.72023	19.06402	86	129	72	1089	175	-4	179	89	71	100	71	1169	230	17	85	646	62	337	62	3626
458	Pinus_hartwegii	-99.70458	19.12211	97	133	71	1237	188	1	187	102	85	112	79	1103	211	16	84	603	65	318	66	3480
459	Pinus_hartwegii	-98.67706	19.18803	73	136	73	1111	166	-19	185	79	58	87	58	958	185	7	88	530	24	313	40	3728
460	Pinus_hartwegii	-98.67259	19.24166	80	140	73	1145	175	-16	191	87	65	94	64	971	185	7	88	539	25	316	39	3653
461	Pinus_hartwegii	-98.6547	19.26177	94	148	73	1300	193	-8	201	103	76	109	76	1010	189	9	87	562	29	327	39	3429
462	Pinus_hartwegii	-98.68489	19.17462	82	141	73	1161	178	-14	192	89	67	96	66	993	190	7	88	554	25	321	39	3632
463	Pinus_hartwegii	-98.63345	19.05508	64	132	74	984	153	-24	177	68	50	76	50	1031	202	7	88	571	27	331	45	3912

464	Pinus_hartwegii	-97.31553	19.02491	62	129	73	1032	153	-23	176	63	56	74	48	1821	346	34	81	989	105	210	155	3865
465	Pinus_hartwegii	-97.30455	19.05332	63	130	73	1038	154	-22	176	65	57	76	49	1807	344	34	81	983	105	209	153	3703
466	Pinus_hartwegii	-97.27966	19.06863	54	126	73	1014	143	-28	171	55	49	67	41	1899	358	37	80	1025	114	218	169	4162
467	Pinus_hartwegii	-97.27088	19.08975	62	129	74	1055	152	-22	174	64	56	75	48	1840	349	36	81	1001	110	210	159	3808
468	Pinus_hartwegii	-97.27088	19.97755	213	109	56	2828	306	114	192	236	202	242	172	2899	488	107	54	1293	349	892	376	3647
469	Pinus_hartwegii	-97.28918	18.98119	71	133	73	1080	163	-17	180	72	65	84	56	1775	341	33	82	973	101	202	147	3800
470	Pinus_hartwegii	-97.32798	19.00888	68	132	73	1050	161	-18	179	69	62	80	53	1748	334	32	82	955	99	203	143	3501
471	Pinus_hartwegii	-97.33456	18.99577	82	138	73	1138	178	-10	188	84	75	95	66	1597	309	28	83	881	88	190	121	3430
472	Pinus_hartwegii	-97.32871	18.97828	66	132	73	1066	159	-20	179	68	61	79	52	1776	339	32	81	968	100	206	147	3328
473	Pinus_hartwegii	-97.27747	18.97901	72	134	74	1106	166	-15	181	74	67	86	57	1766	341	33	82	971	101	200	145	3612
474	Pinus_hartwegii	-97.30089	18.96881	63	130	73	1018	154	-22	176	65	58	75	49	1831	349	34	81	996	105	210	155	3623
475	Pinus_hartwegii	-97.26869	19.07008	51	124	73	985	139	-30	169	52	46	64	38	1932	362	38	79	1039	117	222	175	4131
476	Pinus_hartwegii	-97.27235	19.07955	62	128	73	1055	153	-22	175	63	56	75	48	1839	349	35	81	1000	109	210	159	3957
477	Pinus_hartwegii	-97.24965	19.08611	72	132	73	1097	165	-15	180	74	66	85	57	1764	340	34	82	971	104	445	146	3650
